# Prmt6 represses the pro-adipogenic Ppar-gamma–C/ebp-alpha transcription factor loop

**DOI:** 10.1038/s41598-024-57310-9

**Published:** 2024-03-20

**Authors:** Mirjam Gerstner, Vivien Heller, Johannes Fechner, Benedikt Hermann, Lei Wang, Joern Lausen

**Affiliations:** Department of Eukaryotic Genetics, Institute of Biomedical Genetics, University of Stuttgart, Allmandring 31, 70569 Stuttgart, Germany

**Keywords:** Epigenetics, Eukaryote, Gene expression, Gene regulation

## Abstract

The feed-forward loop between the transcription factors Ppar-gamma and C/ebp-alpha is critical for lineage commitment during adipocytic differentiation. Ppar-gamma interacts with epigenetic cofactors to activate *C/ebp-alpha* and the downstream adipocytic gene expression program. Therefore, knowledge of the epigenetic cofactors associated with Ppar-gamma, is central to understanding adipocyte differentiation in normal differentiation and disease. We found that Prmt6 is present with Ppar-gamma on the *Ppar-gamma* and *C/ebp-alpha* promoter. It contributes to the repression of *C/ebp-alpha* expression, in part through its ability to induce H3R2me2a. During adipocyte differentiation, Prmt6 expression is reduced and the methyltransferase leaves the promoters. As a result, the expression of *Ppar-gamma* and *C/ebp-alpha* is upregulated and the adipocytic gene expression program is established. Inhibition of Prmt6 by a small molecule enhances adipogenesis, opening up the possibility of epigenetic manipulation of differentiation. Our data provide detailed information on the molecular mechanism controlling the Ppar-gamma–C/ebp-alpha feed-forward loop. Thus, they advance our understanding of adipogenesis in normal and aberrant adipogenesis.

## Introduction

The differentiation of mesenchymal stroma cells (MSC) into distinct cell lineages such as adipocytes and osteocytes is accompanied by the timely establishment of cell-type specific gene expression. Adipogenesis and osteogenesis are in a tightly controlled balance and disturbance of this equilibrium is the cause of bone-related disease. Notably, progenitor cells have the ability to differentiate into either the adipocytic or the osteocytic lineage. Remarkably, the competing gene expression programs are initially repressed until cell-type specific transcription factors initiate the respective differentiation program.

C/ebpβ Pparγand C/ebpα are key transcription factors in early adipogenesis. They regulate each other and recruit epigenetic cofactors to their target genes to drive adipogenesis. However, in progenitor cells, they can also exert a repressive function in conjunction with epigenetic cofactors, such as histone deacetylases (HDACs) and protein arginine methyltransferases (PRMTs)^1–7^.

Recently, a role for protein arginine methyltransferase 6 (PRMT6) in the repression of the erythroid gene expression program in hematopoietic progenitor cells has been described^8–10^. Prmt6 might have a similar function in adipocytic progenitors as it associates with Ppar γ, a key transcription factor in early adipogenesis^3^. The methyltransferase can methylate arginine residues in non-histone and histone proteins. The ability of Prmt6 to methylate arginine-2 of histone-3 (H3R2me2a) is well studied, it represses gene expression by counteracting the establishment of the activating H3K4me3 mark^11–14^. However, it can also perform context-dependent activating functions^15–17^. Prmt6 is recruited to target genes by site-specific transcription factors, such as RUNX1, the estrogen receptor, the androgen receptor, NF-kB^9,17–19^ and Pparγ^3^.

The notion that Prmt6 is associated with Pparγon the adipocytic *Fabp4* gene led us to investigate the repressive function of the Prmt6/Pparγgene regulatory complex. The transcription factors Pparγand C/ebpα form a feed-forward loop in adipogenesis^20–24^. However, this feed-forward loop is repressed in progenitor cells by an unknown mechanism. The observation that Pparγand Prmt6 are associated^3^, suggests that the corepressor activity of Prmt6 has a role in controlling the activity of the regulatory loop between Pparγand C/ebpα.

Here, we investigate the impact of Prmt6 on the cross-regulation between Pparγand C/ebpα during adipogenesis. Our data show that Prmt6 is a corepressor of Ppar γ, regulating its own gene expression and the *C/ebp*α expression. In particular, Prmt6 induces the repressive epigenetic marking H3R2me2a at the *C/ebp*α locus. In this way, Prmt6 represses the feed-forward-loop between Pparγand C/ebpα and keeps adipogenesis in check. Upon differentiation, Prmt6 leaves the promoter, repression is released and the proadipogenic feed-forward loop is initiated. Our results provide novel insight into how adipogenesis is controlled at the epigenetic level. This finding may be valuable for the development of epigenetic substances to influence differentiation in a therapeutic setting.

## Materials and methods

### Cell culture

HEK293T cells (DSMZ, Braunschweig, Germany) were cultured in DMEM GlutaMAX medium (Gibco, Waltham, MA, USA), ST2 cells (DSMZ)^25^ in RPMI 1640 GlutaMAX medium (Gibco) supplemented with 10% fetal calf serum (FCS) and 1% Penicillin/Streptomycin (Gibco). The cells were cultured at 37 °C in a 5% CO_2_ atmosphere. At regular intervals, the cell culture was tested as mycoplasma-free. For Prmt6 inhibition, the selective inhibitor SGC6870 (Sigma, Darmstadt, Germany) and the control compound SGC6870N were used in a concentration of 5 µM^26^. For Prmt6 overexpression and knockout experiments, ST2 cells were transduced with Prmt6-LeGOiG2 or gRNA’s in lentiCRISPRv2 vector. Generation and production of virus was done as described^27^. LeGOiG2 empty vector and a vector expressing non-specific gRNA served as controls, respectively. The gRNA sequences are listed in the Supplemental Material Table 1.

### Immunofluorescence and microscopy

ST2 cells were grown on collagen-coated coverslips. Subsequently, they were washed with PBS, fixed with 4% formaldehyde for 10 min at room temperature and washed. Permeabilization was performed by incubation with 0.1% Triton-X 100 (Carl Roth, Karlsruhe, Germany) in PBS for 5 min. Blocking was achieved using 5% BSA in PBS for 30 min. The cells were stained with primary antibodies, washed, and incubated with labeled secondary antibodies. Nuclei were stained with DAPI, cytoplasmatic F-Actin was stained with labelled Phalloidin (iFluor555, Abcam, Amsterdam, Netherlands). The cells were mounted with Fluoramount-G (Invitrogen, Waltham, MA, USA) and analyzed using an LSM710 (Zeiss, Oberkochen, Germany). Antibodies are given in the Supplemental Material Table 8.

### Adipogenic differentiation and Oil-Red-O Staining

ST2 cells^25^ were seeded at a density of 15,000 cells per cm^2^ and grown in adipogenic differentiation medium (DMEM GlutaMAX supplemented with 9% FCS, 250 nM dexamethasone (Sigma-Aldrich, Darmstadt, Germany), 450 µM IBMX (Santa Cruz Biotechnology, Dallas, Texas, USA), 1 µM rosiglitazone (Sigma; Darmstadt, Germany), 5 µg/ml human insulin (Gibco, Waltham, MA, USA). After adipogenic induction, cells were washed with PBS and fixed in 4% formaldehyde for 10 min. Subsequently, cells were rinsed with 60% isopropanol and the wells were dried prior to the addition of Oil-Red-O (Sigma-Aldrich, Darmstadt, Germany). The lipid droplet formation was analyzed by microscopy. For quantification, the Oil-Red-O was extracted from the stained cells with 100% isopropanol and the absorbance was analyzed at 500 nm in a plate reader (Tecan Group Ltd., Maennedorf, Switzerland).

### Chromatin immunoprecipitation (ChIP) assay

Chromatin immunoprecipitation (ChIP) assays were performed according to the X-ChIP protocol from Abcam. DNA purification was done with the ChIP DNA Clean & Concentrator Kit (Zymo Research, Freiburg, Germany). All experiments were performed with at least two different chromatin preparations. Oligonucleotides used for ChIP-qPCR and antibodies are given in the Supplemental Material Tables 4 and 7.

### Co-immunoprecipitation (Co-IP)

HEK293T cells were transfected with 6 µg DNA (Flag-PRMT6-pcDNA3.1, PPARγ1-pcDNA3, PPARγ2-pcDNA3, pcDNA3 empty vector). Whole cell extracts were prepared using lysis buffer [50 mM Tris–HCl (pH 7.5), 150 mM sodium chloride, 10 mM sodium fluoride, 20 mM ß-glycerophosphate, 1% IGEPAL and 1 mM EDTA supplemented with protease inhibitor cocktail (Carl Roth, Karlsruhe, Germany) and 100 mM sodium orthovanadate. After incubation for 10 min, 7.5 mM Magnesium Chloride and 50 U Benzonase were added. The lysate was then incubated on a rotating wheel for 2 h at 4 °C followed by centrifugation at 14.000 rpm for 10 min at 4 °C. Protein concentration was determined by Bradford assay (Bio-Rad Laboratories, Feldkirchen, Germany). 600 µg whole cell lysate and 50 µl Pierce Anti-DYKDDDDK magnetic Agarose-Beads (Thermo Fisher Scientific: Waltham, Massachusetts, USA) were used per IP. The reactions were incubated for 2 h on a rotating wheel at 4°C. The beads were washed twice with lysis buffer and subsequently boiled in 2 × Laemmli buffer for 5 min. The supernatant was applied to a 10% SDS-PAGE and analyzed by immunoblotting.

### Reverse transcription–quantitative polymerase chain reaction (RT-qPCR)

RNA was extracted using the Quick-RNA Miniprep Kit (Zymo Research, Freiburg, Germany) according to the manufacturer’s instruction. cDNA was synthesized with 1 µg RNA using the First-Strand cDNA Synthesis Kit (Invitrogen). qPCR amplification was performed using 1 µl cDNA, 10 µl SYBR Green Mix with ROX additive (Biozym Scientific, Oldendorf, Germany), 1 µl primer mix (10 µM) and 8 µl RNase-free water. Data analysis was performed according to the ΔΔCT method. TBP expression was used as housekeeping gene. Oligonucleotides used for the PCR are given (Supplemental Material Table 2 and 3).

### Immunoblotting

Protein samples were separated by SDS-PAGE and transferred with a wet blot system (Bio-Rad Laboratories, Feldkirchen, Germany) using standard techniques. Primary antibodies are listed in the Supplemental Material Tables 5 and 6.

### Histone extraction

ST2 cells were harvested and washed with ice-cold PBS. For histone extraction, 10^7^ cells/ml were resuspended in Triton extraction buffer (PBS containing 0.5% Triton X 100 (v/v), 2 mM phenylmethylsulfonyl fluoride (PMSF), 0.02% (w/v) NaN_3_). Cells were lysed for 10 min on ice with gentle stirring. After a centrifugation at 650 × g for 10 min at 4 °C, the pellet containing nuclei was washed with half the volume of buffer. At a density of 4 × 10^7^ nuclei/ml the Pellet was resuspended in 0.2N HCl. Histones were acid extracted over night at 4°C. To remove debris, samples were centrifugated as before. The supernatant was neutralized with 2 M NaOH at 1/10 of the volume. Histone lysates were stored at − 80 °C.

### GST-pulldown

500 ng plasmid-DNA of PPARγ2-pcDNA3(2HA) was used in the TNT T7 Quick coupled transcription/translation system (Promega GmbH: Walldorf, Germany), supplemented with^35^S-Methionine (10mCi/ml, 1000Ci/mmol; Hartmann Analytic, Braunschweig, Germany). 10 µl of the in vitro translation was incubated with either GST or GST-PRMT6 beads for 3 h at 4 °C on a rotating wheel^27^. After centrifugation at 2300 rpm for 1 min the pellet was lysed followed by a 10 min incubation at 4 °C on a rotating wheel. This was repeated twice. Samples were denatured at 95 °C with 1.5 × Laemmli buffer. Proteins were separated by SDS-PAGE. The gel was stained with Coomassie-blue. The dried gel was analyzed by phosphorimaging.

### Statistics

Experiments were performed in at least three independent replicates. Results were analyzed using the GraphPad Prism software. Data are presented as mean ± standard error. Statistical significance was calculated using Student’s *t*-test or ANOVA (analysis of variance). As post-hoc statistical test the Holm-Šídák test was used.

## Results

### Expression of Prmt6 during adipogenesis

**Figure 1:**
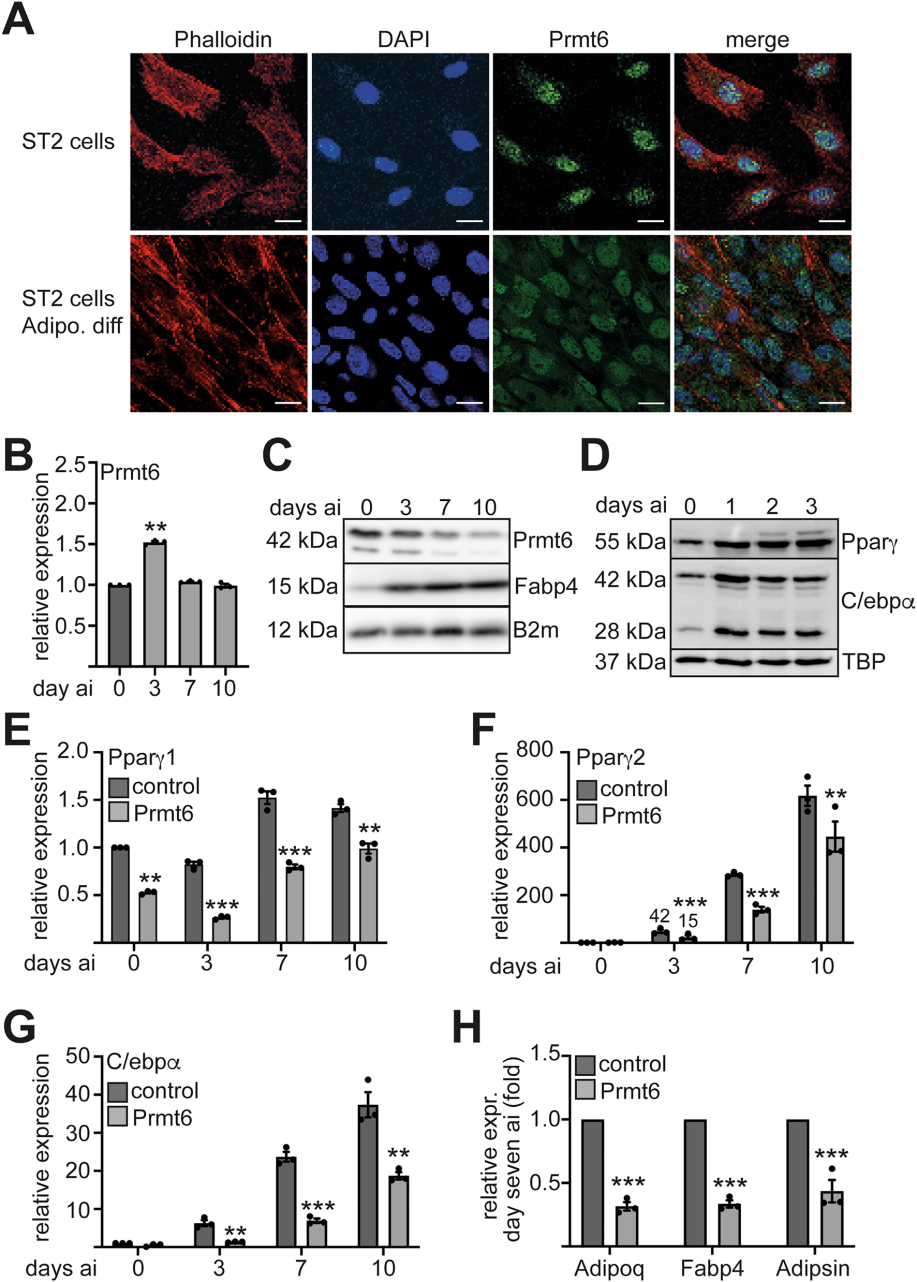
Prmt6 expression during adipocyte differentiation. (**A**) Immunofluorescence staining of Prmt6 in wild type ST2 cells and upon 14 days of adipocyte differentiation. Phalloidin-iFluor555 staining labels the cytoplasmatic F-Actin (red) and blue DAPI staining indicates the nucleus. Prmt6 was detected with an anti-Prmt6 antibody and detected with an Alexa-488 secondary antibody (green). The far-right part of the figure shows the merge of the three signals. Staining indicated the presence of Prmt6 in the nucleus before and after 14 days of adipocytic differentiation. The scale bar indicates 20 µm. The corresponding negative control is shown in the Supplemental Figure S1A. (**B**) Expression of *Prmt6* was determined by qRT-PCR at the indicated time points upon differentiation. (**C**) Western Blot (WB) analysis indicates Prmt6 and Fabp4 protein expression during adipocyte differentiation. WB was performed with lysates of differentiated ST2 cells at the indicated time points and with antibodies against the shown proteins. WB with an anti B2m antibody served as a loading control. (**D**) WB analysis indicates Pparγ and C/ebpα protein expression during adipocyte differentiation. WB was performed with lysates of differentiated ST2 cells at the indicated time points and antibodies against the shown proteins. WB with an anti TBP antibody served as a loading control. (**E**–**H**) Adipocytic gene expression upon Prmt6 overexpression. (**E**) Gene expression of *Ppar*γ*1* was determined by qRT-PCR at the indicated time points upon differentiation in control cells and in Prmt6 expressing cells. (**F**) Gene expression of *Ppar*γ*2* was determined by qRT-PCR at the indicated time points upon differentiation in control cells and in Prmt6 expressing cells. (**G**) Gene expression of *C/ebp*α was determined by qRT-PCR at the indicated time points upon differentiation in control cells and in Prmt6 expressing cells. (**H**) Prmt6 overexpression led to a decreased expression of the marker genes for adipocytes: *Adipoq*, *Fabp4* and *Adipsin*. Expression was determined by qRT-PCR at the seven days of differentiation in control cells and in Prmt6 expressing cells. qRT-PCR was performed with gene specific primer pairs. Data are shown as relative expression normalised to the expression of the housekeeping gene *TBP*, values from day zero were set as one. The error bars display the standard error from the mean of three experiments. The *P*-values were calculated using ANOVA. **P < 0.002, ***P < 0.001.

To examine the localization of Prmt6 during adipogenesis we performed immunofluorescence staining for Prmt6. Prmt6 was present in the nucleus of undifferentiated ST2 cells and with lower staining intensity upon 14 days of adipocyte differentiation (Fig. 1A, Supplemental Figure S1). Interestingly, *Prmt6* expression at the mRNA level was transiently increased during differentiation (Fig. 1B, Supplemental Figure S2, 3), whereas Prmt6 expression was reduced at the protein level consistent with the immunofluorescence data (Fig. 1C). In agreement with the notion that Prmt6 was down regulated during adipogenesis, we found relatively low Prmt6 levels in adipocytic cells in publicly available single cell datasets (Supplemental Figure S4). The adipocytic master regulators Pparγand C/ebpα were upregulated during adipogenesis (Fig. 1D) and adipocyte markers were increased (Supplemental Figure S5, 6).

To examine if the downregulation of Prmt6 is crucial for adipogenesis, we overexpressed Prmt6 (Supplemental Figure S7). Prmt6 expression led to reduced lipid droplet formation (Supplemental Figure S8) compared to the control. Analysis of the *Ppar*γisoforms revealed that *Pparγ1* expression was reduced in the uninduced state and remained lower in Prmt6 expressing cells upon induction of adipogenesis (Fig. 1E). Importantly, the expression of the adipocyte-specific isoform *Ppar*γ*2* was decreased upon differentiation in Prmt6 overexpressing cells (Fig. 1F). Furthermore, *C/ebp*α expression was reduced (Fig. 1G). Consequently, the expression of the downstream adipocytic genes *Adipoq* (Adiponectin), *Fabp4* and *Adipsin*, was lower in Prmt6 expressing cells (Fig. 1F). Taken together, Prmt6 expression was decreased during adipogenesis. Adipogenesis and the expression of the key regulators Pparγ and C/ebpα were reduced upon Prmt6 overexpression.

### Loss of Prmt6 increases C/ebpα and Pparγ expression

**Figure 2:**
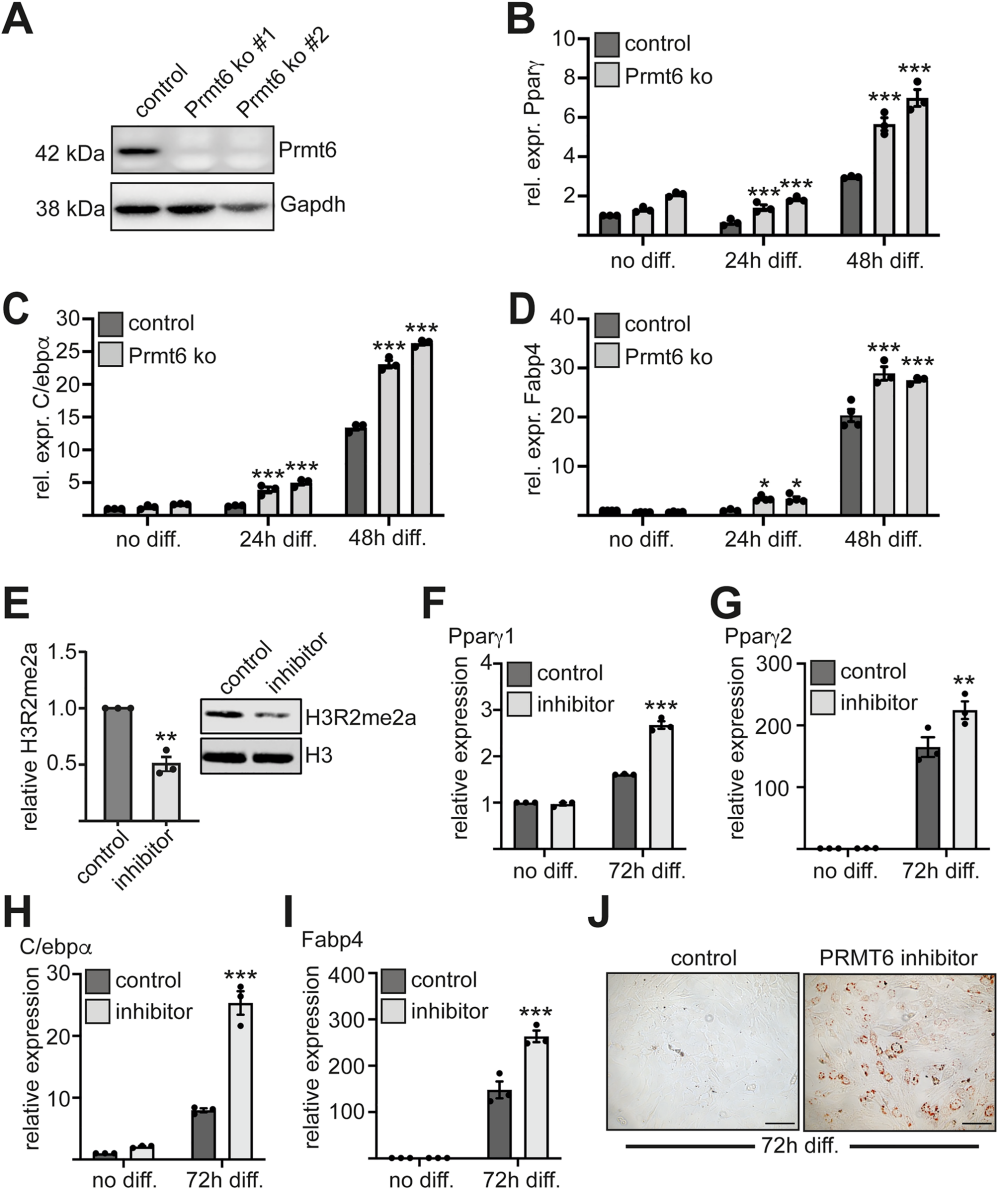
Loss of Prmt6 augments adipocyte differentiation. (**A**) WB displays loss of Prmt6 expression upon CRISPR/Cas9 mediated knockout. Two distinct knockout gRNAs were chosen (ko#1, ko#2). WB was performed with an anti Prmt6 antibody and an anti Gapdh antibody as loading control. (**B**) Gene expression of *Ppar*γ was determined by qRT-PCR at the indicated time points upon differentiation in control cells and in Prmt6 knockout cells. (**C**) Gene expression of *C/ebp*α was determined by qRT-PCR at the indicated time points upon differentiation in control cells and in Prmt6 knockout cells. (**D**) Gene expression of *Fabp4* was determined by qRT-PCR at the indicated time points upon differentiation in control cells and in Prmt6 knockout cells. (**E**) Confirmation of inhibitor activity. WB of histone extracts from control and inhibitor treated ST2 cells after 72h of differentiation was performed with antibodies against H3R2me2 and histone H3. Shown are the values from three determinations of inhibitor treated cells compared to the control (left) and a representative WB (right). The error bars display the standard error of the mean from three independent experiments. The *P*-values were calculated using Student’s t-test comparing control cells with Prmt6 inhibitor treated cells. **P < 0.002. (**F**) Expression of the *Pparγ1* isoform upon inhibition of Prmt6 after 72h of adipocyte differentiation. Gene expression of *Ppar*γ*1* was determined by qRT-PCR at the indicated time points upon differentiation cells treated with Prmt6 inhibitor or control compound. (**G**) Expression of the *Ppar*γ*2* isoform upon Prmt6 inhibition at the mRNA level as determined by qRT-PCR. (**H**) Expression of *C/ebp*α at the mRNA level upon Prmt6 inhibition and adipocyte induction measured by qRT-PCR. (**I**) Gene expression of *Fabp4* in the Prmt6 inhibited cells compared to the control upon 72h of differentiation. (**J**) Inhibition of Prmt6 promotes lipid droplet formation during adipogenesis. The scale bar indicates 100 µm. (**E**–**J**) 5 µM of the specific Prmt6 inhibitor SGC6870 and its control compound SGC6870N was used for Prmt6 inhibition. The qRT-PCR values are shown as relative expression normalised to *TBP* expression. Values gathered for the untreated non-differentiated cells were set as one. The error bars display the standard error of the mean from three independent experiments. The *P*-values were calculated using ANOVA comparing control cells with Prmt6 inhibitor treated cells. **P < 0.002, ***P < 0.001.

Overexpression of Prmt6 suppressed adipogenesis (Fig. 1). To further investigate the influence of Prmt6 on C/ebpα and Pparγ expression Prmt6 was knocked out using CRISPR/Cas9 (Fig. 2A, Supplemental Figure S9). Loss of Prmt6 resulted in an increased Pparγ expression during differentiation (Fig. 2B). Furthermore, C/ebpα expression was increased (Fig. 2C). The expression of the adipocyte marker gene *Fabp4* was augmented in the Prmt6 knockout cells (Fig. 2D).

Subsequently, we used the Prmt6 inhibitor SGC6870 to inhibit endogenous Prmt6 activity. Prmt6 inhibition reduced H3R2me2 (Fig. 2E, Supplemental Figure S9). Furthermore, Prmt6 inhibition resulted in increased *Ppar*γ*1* and *Ppar*γ*2* expression (Fig. 2F, G) and to a pronounced increase in *C/ebp*α expression (Fig. 2H, Supplemental Figure S10). Accordingly, the adipocyte-specific gene *Fabp4* was augmented upon inhibitor treatment at day three of differentiation (Fig. 2I) and lipid droplets were already visible at day three of differentiation in the treated cells (Fig. 2J). These data suggest that loss of Prmt6 increases the potential for adipocytic differentiation by affecting the expression of *C/ebp*α and *Ppar*γ.

### Interaction of Prmt6 with Pparγ

**Figure 3:**
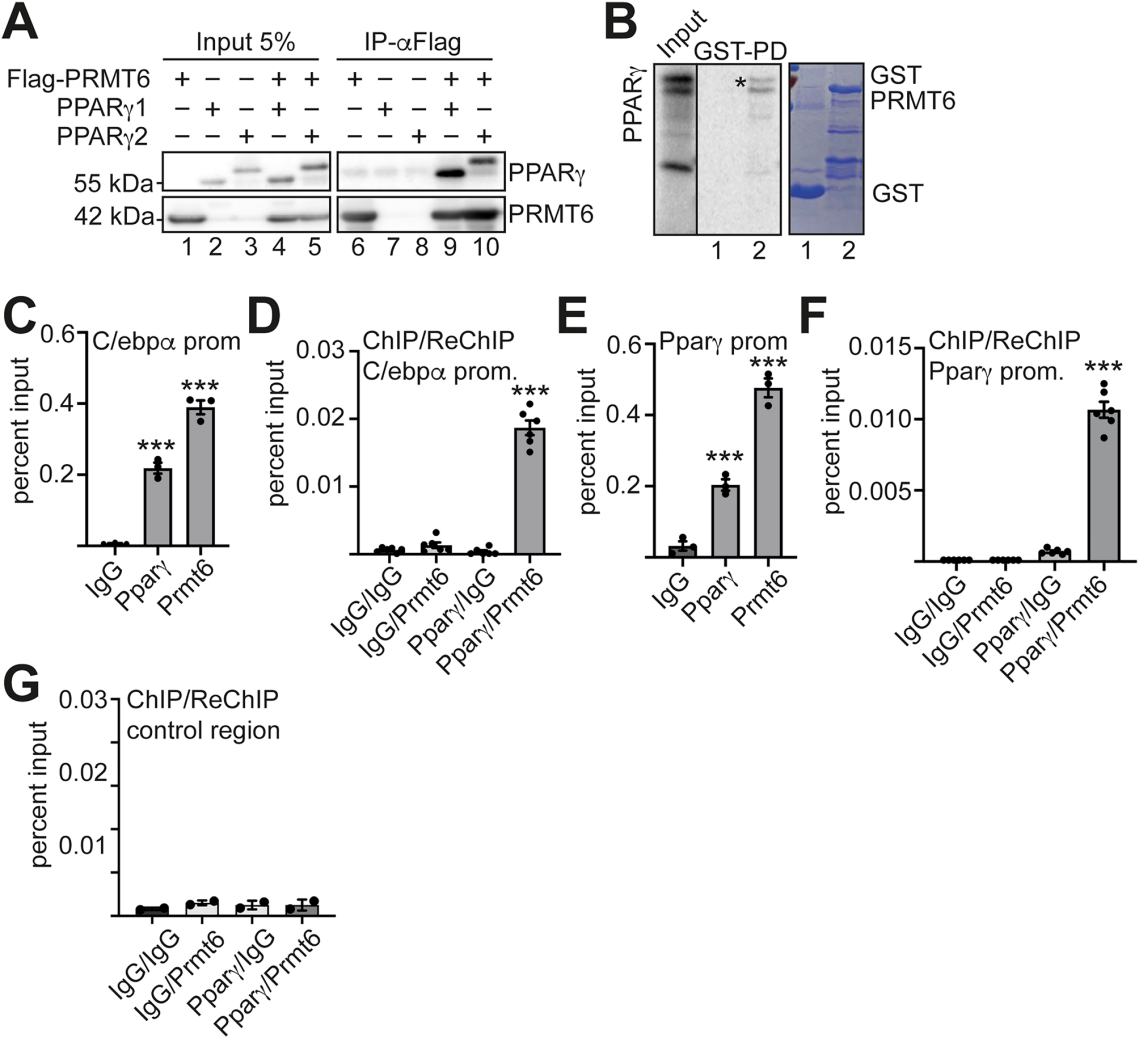
Interaction of Prmt6 with both isoforms of Pparγ. (**A**) To determine the association of PRMT6 with PPARγ, a coimmunoprecipitation was performed in HEK293T cells. Flag-tagged PRMT6 was expressed with either isoform PPARγ1 or PPARγ2. CoIP was done with anti-Flag magnetic beads to pull out Flag-PRMT6. Western blot was performed with anti-Flag antibody to detect Flag-PRMT6 and anti PPARγ antibody. The first five lanes show the input and lanes six to ten shows the IP-fractions. Lane nine and ten shown the coprecipitation of PRMT6 with the PPARγ isoforms, respectively. (**B**) GST-pulldown assay was performed with GST-PRMT6 and in vitro translated^35^S labelled PPARγ. Labelled PPARγ is shown in the input lane on the left. Pull down is shown in the middle and GST input protein is on the right. Pulled out PPARγ is marked by star. (**C**) To evaluate binding of Prmt6 at the *C/ebp*α promoter ChIP was performed with antibodies detecting endogenous Prmt6 and Pparγ, respectively. ChIP was done from undifferentiated ST2 cells. Primer for ChIP qPCR were in the promoter region of *C/ebp*α*.* (**D, F-G**) ChIP-reChIP analysis of Prmt6 and Pparγ binding to target genes. In order to evaluate if Prmt6 and Pparγ bind together to target genes, ChIP-reChIP analysis was performed. (**D**) ChIP-reChIP analysis of Prmt6 and Pparγ at the *C/ebp*α promoter. (**E**) Pparγ and Prmt6 were also present at the *Ppar*γ promoter as determined by ChIP. (**F**) ChIP-reChIP analysis of Prmt6 and Pparγ at the *Ppar*γ promoter. (**G**) ChIP-reChIP analysis of Prmt6 and Pparγ binding at a control region. (**C-**G) ChIP qPCR was performed with loci specific primer pairs. The error bars display the standard error from the mean of at least three experiments. The *P*-values were calculated using ANOVA. ***P < 0.001.

Inhibition of Prmt6 increased the expression of C/ebpα, which is a prominent target gene of Pparγ2 during adipocyte differentiation. This supports the notion that Prmt6 is recruited to the *C/ebp*α promoter by Pparγ^3^. To determine whether Pparγ1 and Ppary2 are able to associate with Prmt6, we performed coimmunoprecipitation (CoIP). CoIP was performed using anti-Flag beads to precipitate Flag-Prmt6. Western blot with an anti-Pparγ antibody showed that both isoforms co-precipitated with Prmt6 (Fig. 3A, Supplemental Figure S11). The interaction of Prmt6 with Pparγ was verified in a GST pull-down experiment with recombinant GST-Prmt6 and in vitro translated S^35^ labeled Pparγ (Fig. 3B, Supplemental Figure S12).

Subsequently, we performed chromatin immunoprecipitation (ChIP) of endogenous Pparγ and Prmt6 at the *C/ebp*α promoter in ST2 cells. We detected Pparγ and Prmt6 close to the transcription start site of the *C/ebp*α gene (Fig. 3C). To determine if Pparγ and Prmt6 concomitantly occupy the *C/ebp*α promoter, we performed ChIP-ReChIP and detected both at the *C/ebp*α promoter (Fig. 3D). Pparγ and PRMT6 were also present at the *Ppar*γ promoter (Fig. 3E, F). At a control region, no binding was detected in ChIP-ReChIP (Fig. 3G). These data demonstrate that Pparγ and Prmt6 are present together at the Pparγ promoter and the *C/ebp*α promotor in the undifferentiated state in ST2 cells.

### Cofactor exchange at the C/ebpα promoter

**Figure 4:**
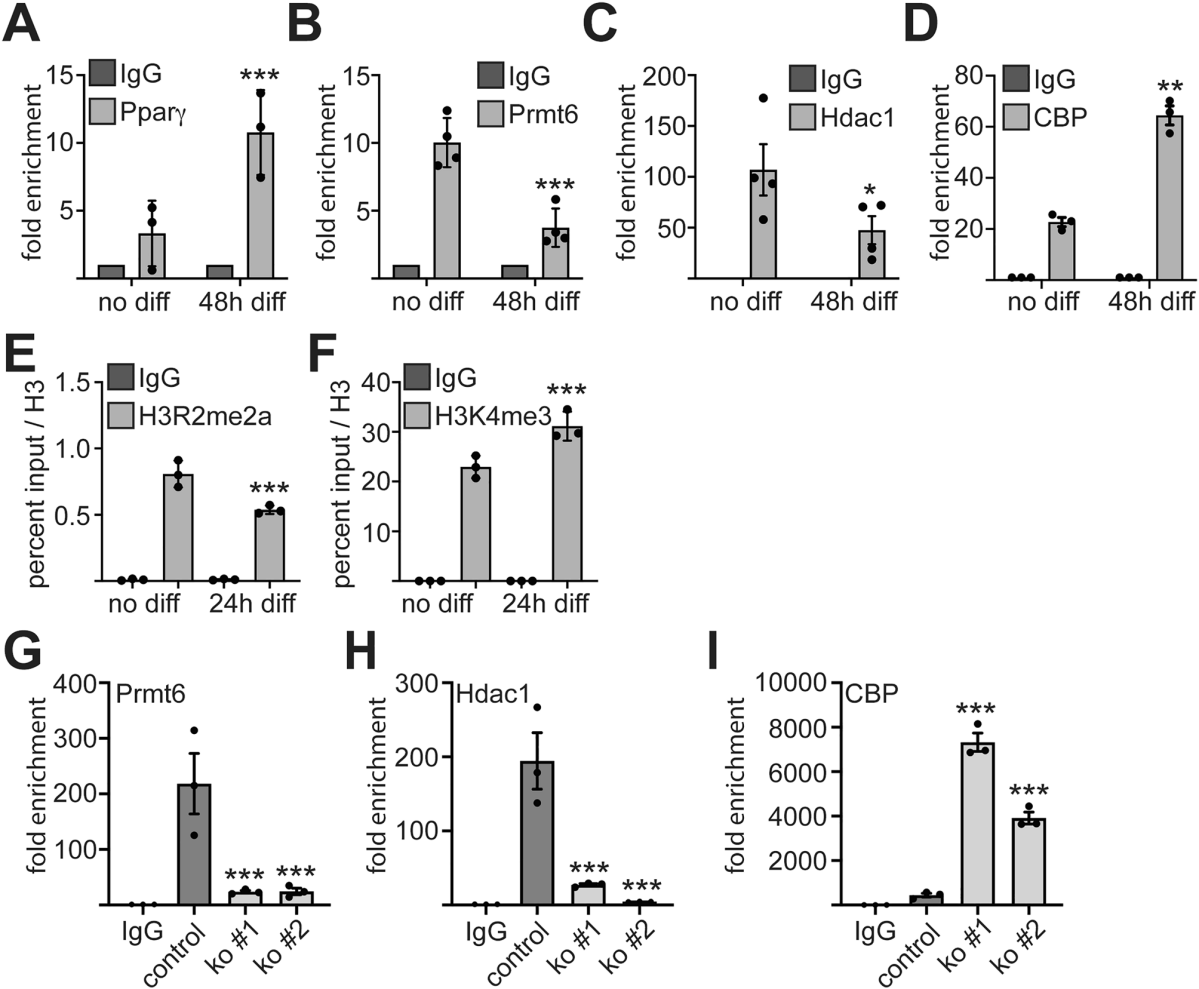
Cofactor exchange at the *C/ebp*α promoter. (**A**–**D**) ChIP analysis of transcription factor binding to the *C/ebp*α promoter in undifferentiated ST2 cells and upon adipocytic differentiation of ST2 cells for 48h. Binding of Pparγ, Prmt6, Hdac1 and CBP to the *C/ebp*α promoter upon adipocytic differentiation is shown. **(E**–**F**) ChIP analysis of histone modifications at the *C/ebp*α promoter in undifferentiated ST2 cells and upon adipocytic differentiation for 24h. (**E**) H3R2me2a at the *C/ebp*α promoter before and after adipocytic differentiation. (**F**) H3K4me3 at the *C/ebp*α promoter before and after adipocytic differentiation. (**G**–**I**) Analysis of the *C/ebp*α locus upon CRISPR/Cas9 mediated Prmt6 knockout. (**G**) Prmt6 binding to the *C/ebp*α promoter upon Prmt6 knockout using an anti Prmt6 antibody. (**H**) Hdac1 binding to the *C/ebp*α promoter upon Prmt6 knockout determined by ChIP. (**I**) Binding of CBP at the *C/ebp*α promoter upon Prmt6 knockout. Data of histone ChIP are given as percent input normalised to an anti-Histone H3 ChIP. Data of transcription factor ChIP are given fold over IgG. The qPCR values were gathered with specific primers against the *C/ebp*α locus. The error bars display the standard error of the mean from at least three independent experiments. The *P*-values were calculated using ANOVA. *P < 0.033, **P < 0.002, ***P < 0.001.

**Figure 5:**
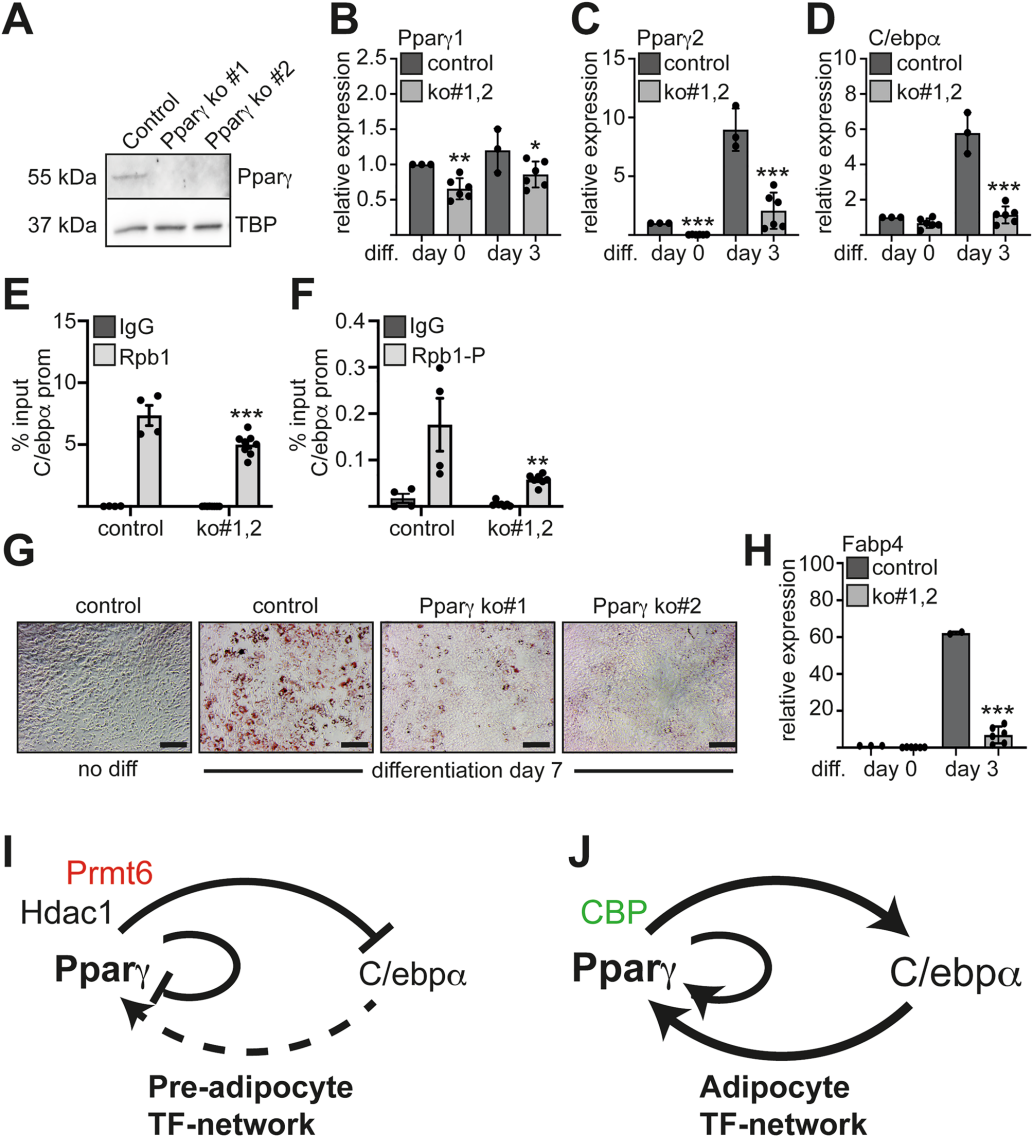
Influence on the Pparγ–C/ebpα feed-forward loop. *Pparγ* knockout was generated by lentiviral transduction of the CRISPR/Cas9 system with two gRNAs in ST2 cells (ko#1 and ko#2). (**A**) Knockout of *Ppar*γ was confirmed by Western Blot using an anti Pparγ antibody. (**B**–**C**) Both isoforms of *Ppar*γ showed a reduced expression upon knockout in undifferentiated cells and upon adipocytic differentiation determined by qRT-PCR. (**D**) *C/ebp*α expression upon differentiation in the *Pparγ* knockout cells as measured by qRT-PCR. (**E**) Rpb1 binding to the *C/ebp*α promoter was reduced upon Pparγ knockout. Rpb1 CTD antibody detects levels of total Rpb1 protein (both phosphorylated and unphosphorylated forms). (**F**) The phosphorylated form of Rpb1 CTD at Serin 2 showed reduced occupancy at the *C/ebp*α promoter upon Pparγ knockout. Data of ChIP are given as percent of input. (**G**) Oil-Red-O staining revealed reduced lipid droplet formation in the *Pparγ* knockout cells compared to control at day 7 of differentiation (N = 6). Representative images of the Oil-Red-O staining are shown. The scale bar indicates 100 µm. (**H**) Expression of the adipocytic gene *Fabp4* in *Ppar*γ knockout cells. The qRT-PCR values are shown as relative expression normalised to TBP expression. Values gathered for the non-differentiated cells were set as one. The error bars display the standard error of the mean from at least three independent experiments. The *P*-values were calculated using ANOVA. *P < 0.033, **P < 0.002, ***P < 0.001. (**I**–**J**) Schematic representation of Prmt6 within the Pparγ–C/ebpα feed-forward loop. (**I**) Pre-adipocytic transcription factor network with low Pparγ and C/ebpα expression, which is repressed by Prmt6 and other corepressors. (**J**) Upon adipocytic differentiation Prmt6 leaves Pparγ occupied promoter and coactivators are recruited. The Pparγ–C/ebpα feed-forward loop is activated.

Our data show that Prmt6 negatively affects the expression of the Pparγ target gene *C/ebp*α and counteracts adipogenesis. Thus, we examined the Pparγ/Prmt6 complex at the *C/ebp*α promoter before and after adipocytic differentiation. As expected, the amount of Pparγ increased upon differentiation (Fig. 4A). Prmt6 binding was reduced (Fig. 4B). Furthermore, binding of the corepressor Hdac1 was reduced in the differentiated cells (Fig. 4C). Concomitantly to reduced Hdac1 amount, binding of the coactivator CBP to the *C/ebp*α promoter was increased (Fig. 4D). At the histone level, H3R2me2a was reduced at the *C/ebp*α promoter upon adipocytic differentiation (Fig. 4E). The activating histone modification H3K4me3 was already present in the uninduced state and increased upon differentiation (Fig. 5F). These data indicate that chromatin at the *C/ebp*α promoter is not in a repressed state in undifferentiated cells. This is consistent with our observation that Pparγ and C/ebpα are expressed to some level in these cells (Fig. 1).

To further analyze the influence of Prmt6 on the state of the *C/ebp*α promoter we knocked out Prmt6 using CRISPR/Cas9. Reduction of Prmt6 was detected at the *C/ebp*α promoter upon knockout (Fig. 4G). In addition, Hdac1 binding was reduced (Fig. 4H) and binding of CBP was increased (Fig. 4I). These data indicate that Prmt6 levels at the *C/ebp*α promoter influence the presence of corepressors and coactivators.

### C/ebpα promoter is poised for a signal from the Pparγ-Cebpα feed-forward loop

We have shown that Prmt6 interacts with Pparγ and is recruited to the *C/ebp*α promoter with Pparγ. To further investigate the function of Pparγ with Prmt6 during adipocytic differentiation, we knocked out Pparγ using CRISPR/Cas9. Upon knockout of Pparγ (Fig. 5A, Supplemental Figure S13), Pparγ was no longer induced upon adipocyte differentiation (Fig. 5B, C). In agreement with the observation that Pparγ is a main activator of *C/ebp*α*,* the increase of C/ebpα expression upon differentiation was also absent in the Pparγ knockout cells (Fig. 5D). RNApolII is present at the C/ebpα promoter in the non-differentiated cells (Fig. 5E). Upon loss of Pparγ expression the presence of RNApolII on the C/ebpα promoter is reduced to some degree (Fig. 5E). However, the phosphorylated form of RNApolII, which is the transcriptionally active form of the enzyme is reduced substantially in the Pparγ knockout cells (Fig. 5F). Consequently, adipocytic differentiation as evaluated by lipid droplet formation was reduced upon *Ppar*γ knockout (Fig. 5G). Accordingly, expression of C/ebpα target gene *Fabp4* was reduced (Fig. 5H). These data indicate that the C/ebpα promoter is in a poised state in before differentiation is executed. Taken together, our data show that Prmt6 is part of a regulatory mechanism which controls the Pparγ-Cebpα feed-forward loop in progenitor cells (Fig. 5I, J).

## Discussion

Adipocytic progenitor cells are in a delicate balance between adipogenic and osteogenic differentiation. In the progenitor population both cell-type specific gene expression programs can be initiated, but are repressed until differentiation is induced. We found that Prmt6 antagonizes the activation of a Pparγ-C/ebpα feed-forward loop, which controls adipogenesis (Fig. 5G, H). This way Prmt6 contributes to repression of the adipogenic gene expression program in progenitor cells.

### Prmt6 is reduced during adipogenesis

Prmt6 is present in the nucleus and is downregulated upon induction towards adipogenesis. Downregulation was detected at the protein level, whereas Prmt6 levels were transiently increased at the mRNA level (Fig. 1). This could indicate posttranscriptional regulation of Prmt6, potentially by an adipocyte associated microRNA. Alternatively, Prmt6 transcript stability could be regulated by RNA-methylation as was recently described for Prmt6^28^. There is also evidence for regulation of Prmt6 by proteasomal degradation^29,30^. In our hands, treatment of proteasome inhibitor MG132 did not lead to an increased Prmt6 protein level in ST2 cells (Supplemental Figure S3). At the pre-adipocyte state of differentiation, Pparγ and C/ebpα are expressed at a low level (Fig. 1).

In case the downregulation of Prmt6 was inhibited by Prmt6 over-expression, the activation of the adipocyte specific transcription factors Pparγ and C/ebpα was reduced. As a consequence, adipocytic gene expression and adipogenesis was reduced. This indicates that the observed downregulation of Prmt6 during adipocyte differentiation has a physiological role. Prmt6 might keep the adipocytic gene expression in check. This way progenitors can maintain the potential to differentiate into distinct cell types and only upon down-regulation the adipocytic potential is released. Thus, it would be interesting to study the role of Prmt6 during osteocyte differentiation, which is in a balance with adipocytic differentiation.

### Loss of Prmt6 augments adipogenesis

Prmt6 has a profound effect on the differentiation of distinct cell types^10,31^ and knockdown of Prmt6 by shRNA increases adipocyte differentiation^3^. Likewise, knockout of Prmt6 by CRISPR/Cas9 led to increased Pparγ and C/ebpα expression. Furthermore, inhibition of Prmt6 by a small-molecule inhibitor augmented adipogenesis (Fig. 2). Prmt6 interacts with Pparγ and is present with the transcription factor on the *C/ebp*α and the *Ppar*γ promoter (Fig. 3). This indicates that Prmt6 influences the Pparγ-C/ebpα circuit at several levels (Fig. 5). Upon induction of adipocyte differentiation, we observed that the amount of Pparγ increased at the *C/ebp*α promoter (Fig. 4). Whereas Prmt6 and Hdac1 are reduced at the *C/ebp*α promoter upon differentiation. Concomitantly, the coactivator CBP is recruited. This observation supports the notion that Prmt6 is a corepressor associated with Pparγ, which is exchanged by coactivating cofactors (Reviewed in^32^). A similar observation has been made by us in case of a RUNX1-PRMT6 complex in megakaryocytic differentiation^9^. Because Pparγ expression and binding to the *C/ebp*α promoter significantly increases upon differentiation, the formation of a novel regulatory complex is likely. Pparγ complex formation might also be influenced by posttranslational modification of the transcription factor. Notably, Pparγ can be methylated by the protein arginine methyl transferase 4 (PRMT4)^33^. However, no evidence was found for methylation of PPARγ by PRMT6^3^.

### ***Prmt6 activity maintains C/ebp***α*** in an activatable state***

We found the activating histone marks H3K4me3 already present in undifferentiated cells at the *C/ebp*α locus. This is in line with the notion, that the formation of an active chromatin environment precedes activation of genes expression during adipocytic differentiation^34–36^. However, the repressive H3R2me2a, which is mediated by Prmt6 is reduced upon differentiation and H3K4me3 is augmented. H3R2me2a counteracts the establishment of H3K4me3. There is an interplay between arginine methyl transferases and lysine demethylases. KDM1A promotes adipocyte differentiation through repressing Wnt signaling, which is important for osteogenic differentiation^37^. As PRMT6 and KDM1A interact^38,39^, it is conceivable that PRMT6 and KDM1A concomitantly regulate the branching between adipogenesis and osteogenesis. Further studies are required to investigate a potentially PRMT6-KDM1A-axis. Loss of the Prmt6 protein does not alter *C/ebp*α expression in the undifferentiated cells but enhances its activation upon induction of differentiation (Fig. 2). Furthermore, the presence of Hdac1 is reduced and CBP is increased upon loss of Prmt6. This could be caused by a role of Prmt6 as a scaffold protein, which contributes to Hdac1 binding. Alternatively, Prmt6 could methylate transcription factors in its proximity and this way alter cofactor recruitment, as has been shown for the transcription factor Runx1 and Prmt1^40^.

### ***Knockout of Ppar***γ*** leads to decreased adipocytic identity***

Pparγ is a central adipocytic lineage-determining factor^41–43^ and it is cooperating with C/ebpα^20,22,44^. We firmly established that Pparγ is present with Prmt6 on its own promoter and the *C/ebp*α promoter. In agreement with the presence of H3K4me3 at the C/ebpα promoter in the non-differentiated state, we also detected RNApolII at the promoter, although expression of C/ebpα was low (Fig. 5). This indicates that the adipocytic gene expression program is in a pre-activated state in progenitor cells. Our data show that loss of Pparγ leads to a breakdown of the adipocytic gene expression program (Fig. 5). These results indicate, that Prmt6 has a role in adipocytic progenitor cells, before the Pparγ–C/ebpα feed-forward loop is activated. It suppresses the Pparγ–C/ebpα feed-forward loop (Fig. 5), but leaves it activatable. Manipulation of this regulatory loop through Prmt6 inhibition could be a route to influence differentiation in a therapeutic setting.

### Supplementary Information

Supplementary Figures.

Supplementary Tables.

## Data Availability

Data are provided within the manuscript and the supplementary information files; this includes full-length blots. Please contact the corresponding author Jörn Lausen (J.L) to request data or material from this study.

## References

[1] LeBlanc SE, Wu Q, Lamba P, Sif S, Imbalzano AN (2016). Promoter-enhancer looping at the PPARgamma2 locus during adipogenic differentiation requires the Prmt5 methyltransferase. Nucl. Acids Res..

[2] LeBlanc SE (2012). Protein arginine methyltransferase 5 (Prmt5) promotes gene expression of peroxisome proliferator-activated receptor gamma2 (PPARgamma2) and its target genes during adipogenesis. Mol. Endocrinol..

[3] Hwang JW, So YS, Bae GU, Kim SN, Kim YK (2019). Protein arginine methyltransferase 6 suppresses adipogenic differentiation by repressing peroxisome proliferatoractivated receptor gamma activity. Int. J. Mol. Med..

[4] Hu YJ (2015). Transcriptional and post-transcriptional control of adipocyte differentiation by Jumonji domain-containing protein 6. Nucl. Acids Res..

[5] Picard F (2004). Sirt1 promotes fat mobilization in white adipocytes by repressing PPAR-gamma. Nature.

[6] Yadav N (2008). CARM1 promotes adipocyte differentiation by coactivating PPARgamma. EMBO Rep..

[7] Gelman L (1999). p300 interacts with the N- and C-terminal part of PPARgamma2 in a ligand-independent and -dependent manner, respectively. J. Biol. Chem..

[8] Kuvardina ON (2015). RUNX1 represses the erythroid gene expression program during megakaryocytic differentiation. Blood.

[9] Herglotz J (2013). Histone arginine methylation keeps RUNX1 target genes in an intermediate state. Oncogene.

[10] Herkt SC (2018). Protein arginine methyltransferase 6 controls erythroid gene expression and differentiation of human CD34(+) progenitor cells. Haematologica.

[11] Guccione E (2007). Methylation of histone H3R2 by PRMT6 and H3K4 by an MLL complex are mutually exclusive. Nature.

[12] Kirmizis A (2007). Arginine methylation at histone H3R2 controls deposition of H3K4 trimethylation. Nature.

[13] Hyllus D (2007). PRMT6-mediated methylation of R2 in histone H3 antagonizes H3 K4 trimethylation. Genes Dev..

[14] Iberg AN (2008). Arginine methylation of the histone H3 tail impedes effector binding. J. Biol. Chem..

[15] Harrison MJ, Tang YH, Dowhan DH (2010). Protein arginine methyltransferase 6 regulates multiple aspects of gene expression. Nucl. Acids Res..

[16] Casadio F (2013). H3R42me2a is a histone modification with positive transcriptional effects. Proc. Natl. Acad. Sci. USA.

[17] Di Lorenzo A, Yang Y, Macaluso M, Bedford MT (2014). A gain-of-function mouse model identifies PRMT6 as a NF-kappaB coactivator. Nucl.ic Acids Res..

[18] Sun Y, Chung HH, Woo AR, Lin VC (1843). Protein arginine methyltransferase 6 enhances ligand-dependent and -independent activity of estrogen receptor alpha via distinct mechanisms. Biochim. Biophys. Acta.

[19] Scaramuzzino C (2015). Protein arginine methyltransferase 6 enhances polyglutamine-expanded androgen receptor function and toxicity in spinal and bulbar muscular atrophy. Neuron.

[20] Lefterova MI (2008). PPARgamma and C/EBP factors orchestrate adipocyte biology via adjacent binding on a genome-wide scale. Genes Dev..

[21] Nielsen R (2008). Genome-wide profiling of PPARgamma:RXR and RNA polymerase II occupancy reveals temporal activation of distinct metabolic pathways and changes in RXR dimer composition during adipogenesis. Genes Dev..

[22] Madsen MS, Siersbaek R, Boergesen M, Nielsen R, Mandrup S (2014). Peroxisome proliferator-activated receptor gamma and C/EBPalpha synergistically activate key metabolic adipocyte genes by assisted loading. Mol. Cell. Biol..

[23] Rauch A, Mandrup S (2021). Transcriptional networks controlling stromal cell differentiation. Nat. Rev. Mol. Cell. Biol..

[24] Tontonoz P, Spiegelman BM (2008). Fat and beyond: The diverse biology of PPARgamma. Annu. Rev. Biochem..

[25] Abdallah BM, Alzahrani AM, Abdel-Moneim AM, Ditzel N, Kassem M (2019). A simple and reliable protocol for long-term culture of murine bone marrow stromal (mesenchymal) stem cells that retained their in vitro and in vivo stemness in long-term culture. Biol. Proced. Online.

[26] Shen Y (2021). A first-in-class, highly selective and cell-active allosteric inhibitor of protein arginine methyltransferase 6. J. Med. Chem..

[27] Schneider L (2021). PRMT6 activates cyclin D1 expression in conjunction with the transcription factor LEF1. Oncogenesis.

[28] Cheng Y (2023). Decoding m(6)A RNA methylome identifies PRMT6-regulated lipid transport promoting AML stem cell maintenance. Cell. Stem Cell..

[29] Chen W (2020). SCF-FBXO24 regulates cell proliferation by mediating ubiquitination and degradation of PRMT6. Biochem. Biophys. Res. Commun..

[30] Li T (2021). F-box protein FBXW17-mediated proteasomal degradation of protein methyltransferase PRMT6 exaggerates CSE-induced lung epithelial inflammation and apoptosis. Front. Cell. Dev. Biol..

[31] Lee YH (2012). Protein arginine methyltransferase 6 regulates embryonic stem cell identity. Stem Cells Dev..

[32] Viswakarma N (2010). Coactivators in PPAR-regulated gene expression. PPAR Res..

[33] Zhong Y (2023). PRMT4 facilitates white adipose tissue browning and thermogenesis by methylating PPARgamma. Diabetes.

[34] Mikkelsen TS (2010). Comparative epigenomic analysis of murine and human adipogenesis. Cell.

[35] Siersbaek R (2011). Extensive chromatin remodelling and establishment of transcription factor 'hotspots' during early adipogenesis. EMBO J..

[36] Sarusi Portuguez A (2017). Hierarchical role for transcription factors and chromatin structure in genome organization along adipogenesis. FEBS J..

[37] Wang D, Kuang YL, Zhang GL, Xiao K, Liu YL (2022). Lysine-specific demethylase 1 in energy metabolism: A novel target for obesity. J. Nutr..

[38] Prakasam R (2023). LSD1/PRMT6-targeting gene therapy to attenuate androgen receptor toxic gain-of-function ameliorates spinobulbar muscular atrophy phenotypes in flies and mice. Nat. Commun..

[39] Musri MM (2010). Histone demethylase LSD1 regulates adipogenesis. J. Biol. Chem..

[40] Zhao X (2008). Methylation of RUNX1 by PRMT1 abrogates SIN3A binding and potentiates its transcriptional activity. Genes Dev..

[41] Hu E, Tontonoz P, Spiegelman BM (1995). Transdifferentiation of myoblasts by the adipogenic transcription factors PPAR gamma and C/EBP alpha. Proc. Natl. Acad. Sci. U.S.A..

[42] Tontonoz P, Hu E, Spiegelman BM (1994). Stimulation of adipogenesis in fibroblasts by PPAR gamma 2, a lipid-activated transcription factor. Cell.

[43] Rosen ED (1999). PPAR gamma is required for the differentiation of adipose tissue in vivo and in vitro. Mol. Cell..

[44] Wu Z (1999). Cross-regulation of C/EBP alpha and PPAR gamma controls the transcriptional pathway of adipogenesis and insulin sensitivity. Mol. Cell..

