# Supplementary material for: Prmt6 represses the pro-adipogenic Ppar-gamma–C/ebp-alpha transcription factor loop
Source: Sci Rep. 2024 Mar 20;14:6656. doi: 10.1038/s41598-024-57310-9 (PMC10954715; doi:10.1038/s41598-024-57310-9)
Supplement: Supplementary file 1 — Supplementary Figures. [file 41598_2024_57310_MOESM1_ESM.pdf]

Supplementary Figures

Gerstner et al.

Supplementary Figure S1
Related to Figure 1

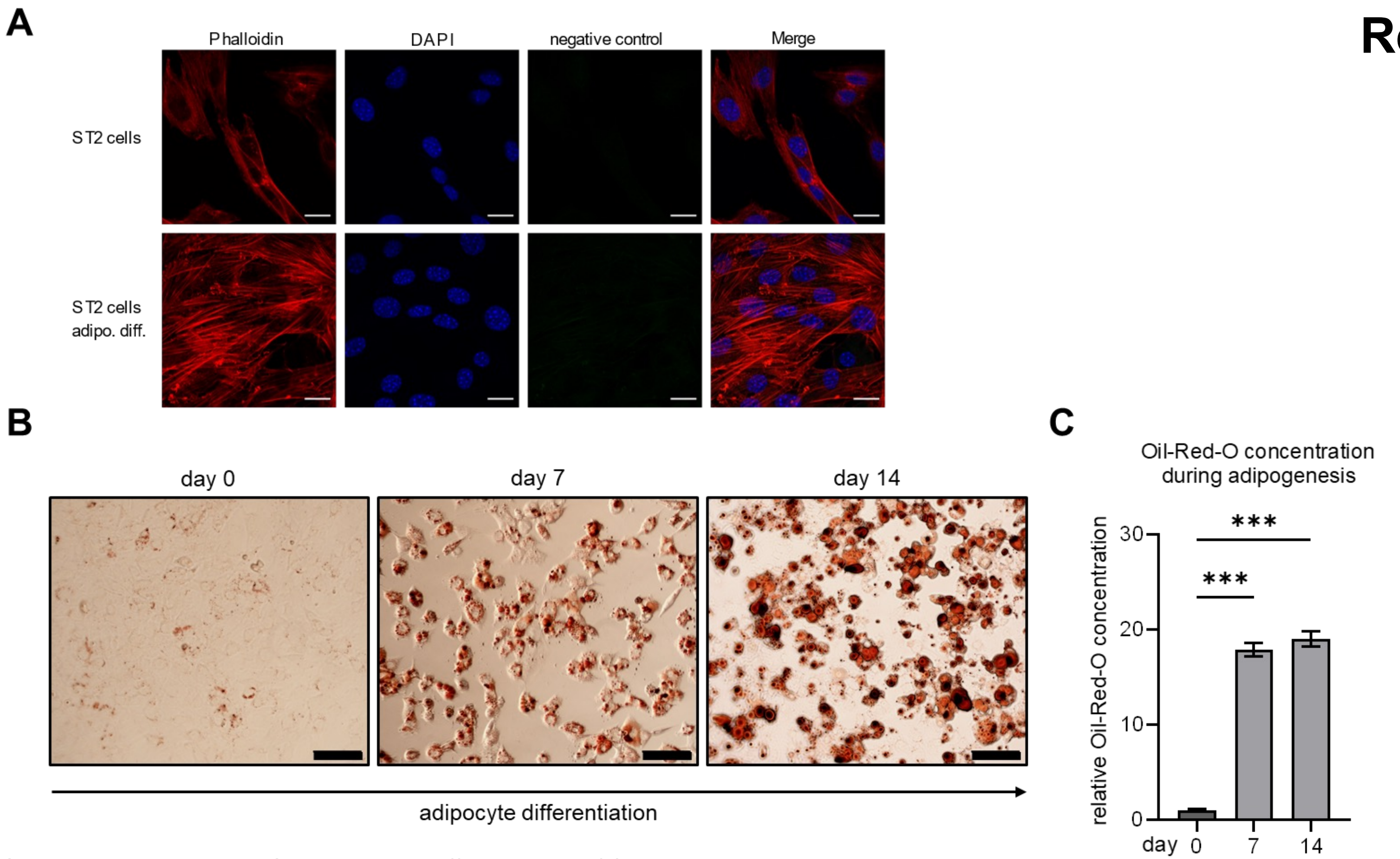

Supplementary Figure S1. Adipocyte differentiation of ST2 cells.
Adipocyte differentiation of ST2 cells was induced with differentiation media for 7 or 14 days. **(A)** Immunofluorescence staining upon adipocyte differentiation. Red phalloidin-iFluor555 staining labels the cytoplasmatic F-Actin and blue DAPI DNA-staining indicates the nucleus. No staining is visible in the negative control. The far-right part of the figure shows the merge of the three signals. The white scale bar indicates 20 µm. **(B)** To validate the successful adipocyte differentiation of ST2 cells Oil-Red-O staining was performed. Cells show an increasing amount of lipid droplets upon treatment with differentiation media for 7 days. This effect is even elevated at day 14 of adipogenesis. The black scale bar indicates 100 µm. **(C)** Quantitative measurement of the relative Oil-Red-O confirmed these findings. Higher Oil-Red-O concentrations were determined in the differentiated cells compared to day 0. The error bars display the standard error from the mean of three experiments. The *P*-values were calculated using ANOVA. ***$P < 0.001$.

Supplementary Figure S2
Related to Figure 1

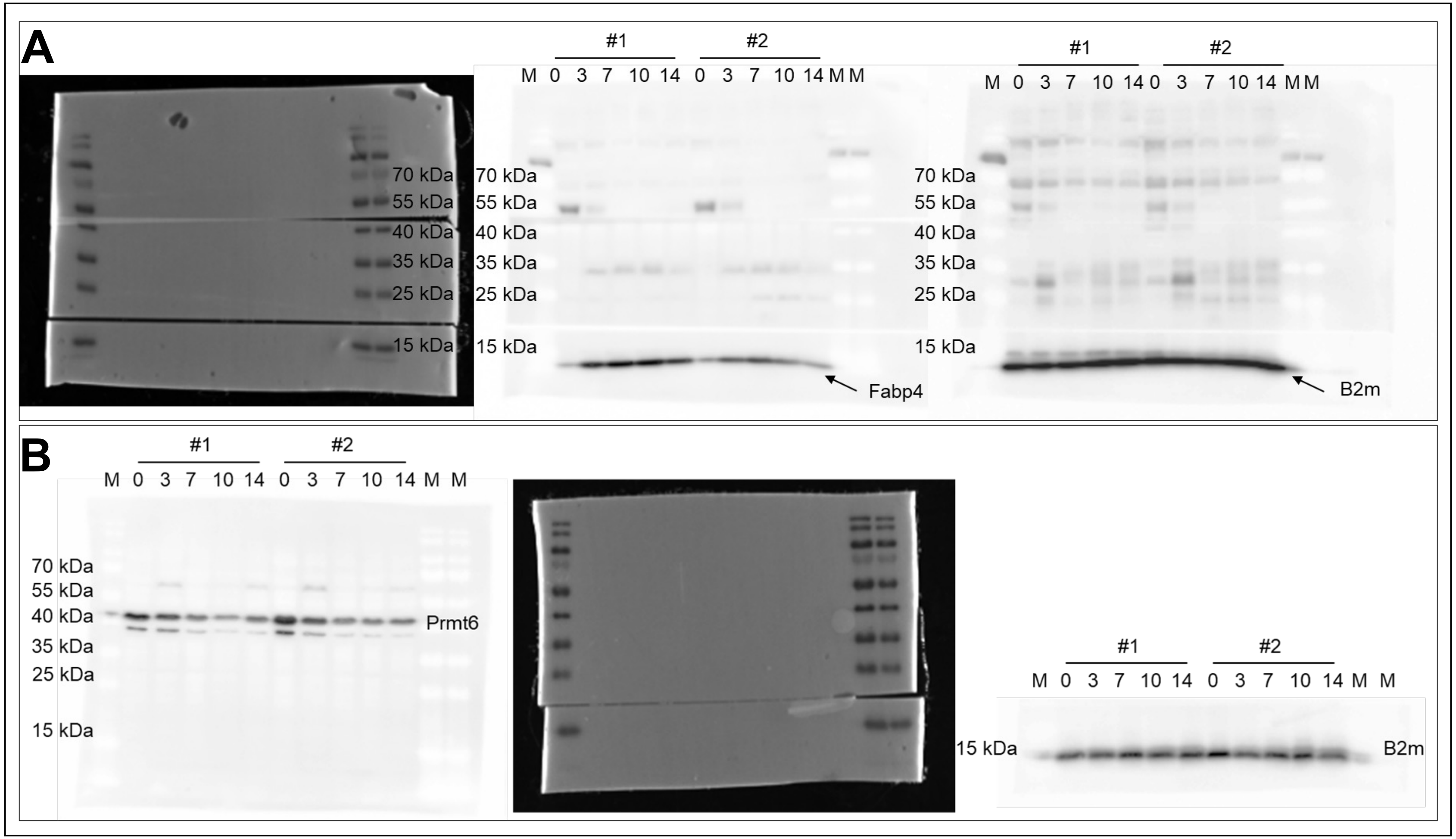

Supplementary Figure S2. Raw data of the Western Blot upon adipogenesis in ST2 cells.
(A-B) Raw data related to the Western Blot shown in **Figure 1C.** Adipocyte differentiation in ST2 cells was induced with differentiation media for 14 days. Western Blot analysis was performed with whole cell lysates of differentiated ST2 cells at various time points. The time points are 0, 3, 7 ,10 and 14 days after induction. **(A)** The membrane was cut into three pieces in order to perform several stainings at the same time. The lowest fragment was stained with Fabp4 (Cell Signaling, #2120). B2m (Cell Signaling, #59035) served as a loading control. **(B)** On another membrane, Prmt6 (Cell Signaling, #14641) was stained. After this staining, the membran was cut into two pieces. The lowest fragment was stained with B2m (Cell Signaling, #59035) as a loading control. **(A-B)** All antibodies were used with a dilution of 1:1000. Secondary antibody anti-rabbit IgG H&L (HRP) (Abcam, ab97080) was used with a dilution of 1:10.000. All antibodies were diluted in 4% milk/TBS-T. For size estimation PageRuler™ (Invitrogen, 26617) was applied.

Supplementary Figure S3
Related to Figure 1

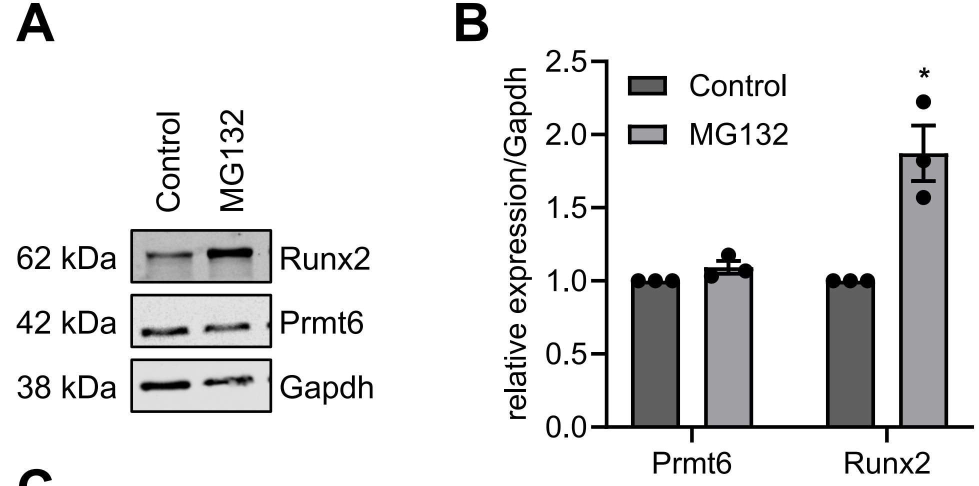

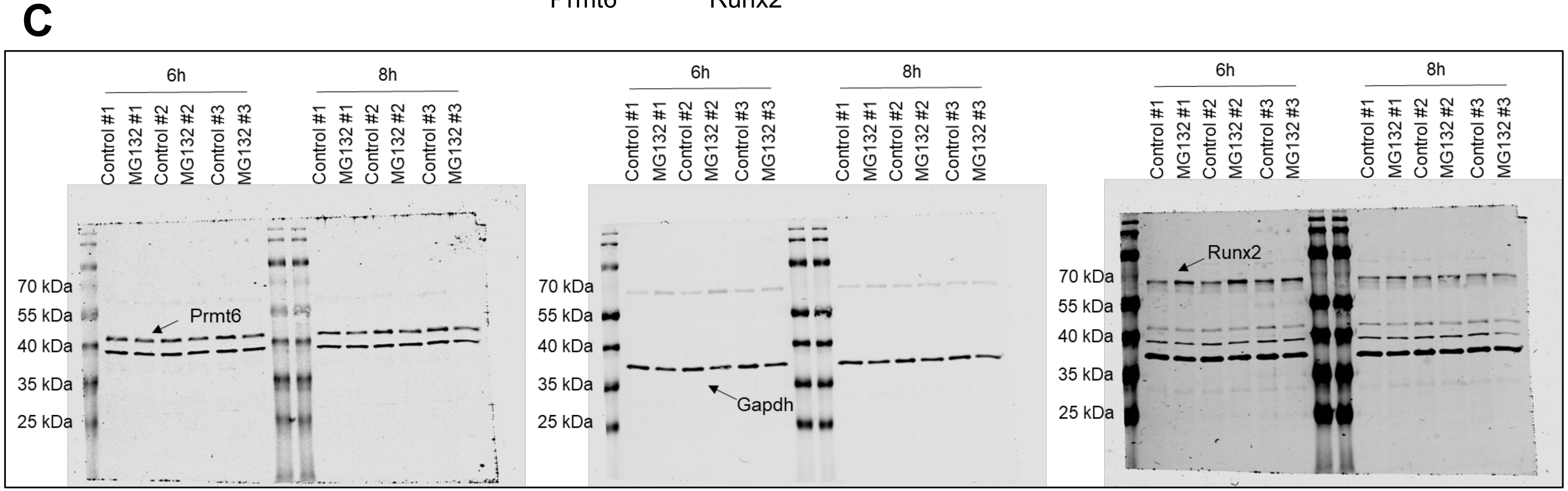

Supplementary Figure S3. Proteasomal degradation of Prmt6 in ST2 cells.

Since Prmt6 decreases at the protein level during adipogenesis, but not at the mRNA level, proteasomal degradation was examined. ST2 cells were treated with either DMSO as a control or 20 µM proteasome inhibitor MG132 (sc-201270, Santa Cruz Biotechnology). After 6 hours of treatment, cells were harvested. **(A)** Western Blot analysis of Prmt6 revealed no increase upon inhibitor treatment. Runx2 was stained as a positive control (Chava et al., 2018) and was increased after proteasome inhibition. **(B)** Protein amounts were quantified with ImageJ and normalized against Gapdh. The error bars display the standard error from the mean of three experiments. The *P*-values were calculated using student's t-test. *$P < 0.033$. **(C)** Raw data of the western blot analysis for proteasomal degradation of Prmt6. Prmt6 (Cell Signaling, #14641) was stained in parallel with Gapdh (Invitrogen, MA5-15738) as a loading control. Afterwards Runx2 (Cell Signaling, #12556) was stained. All primary antibodies were diluted 1:1000 in blocking reagent (Roche #11096176001) in TBS. Secondary antibodies (Li-Cor, 926-68070, 926-32211 and 926-68071) were used at a dilution of 1:20000. PageRuler™ (Invitrogen, 26617) was applied for size estimation. Additional samples of MG132 treatment for 8h were applied to the gel but are not part of figure (A) and (B).

Chava S, Chennakesavulu S, Gayatri BM, Reddy ABM. A novel phosphorylation by AMP-activated kinase regulates RUNX2 from ubiquitination in osteogenesis over adipogenesis. Cell Death Dis. 2018 Jul 9;9(7):754. doi: 10.1038/s41419-018-0791-7 . PMID: 29988028 ; PMCID: PMC6037667.

Supplementary Figure S4

Related to Figure 1

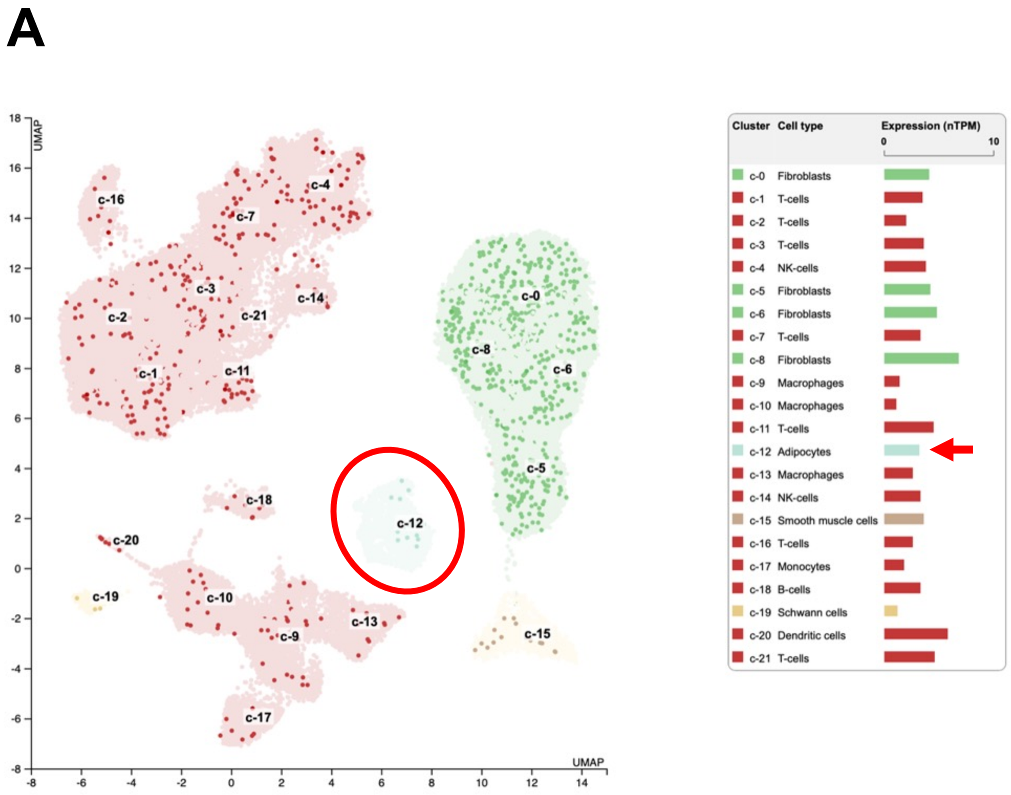

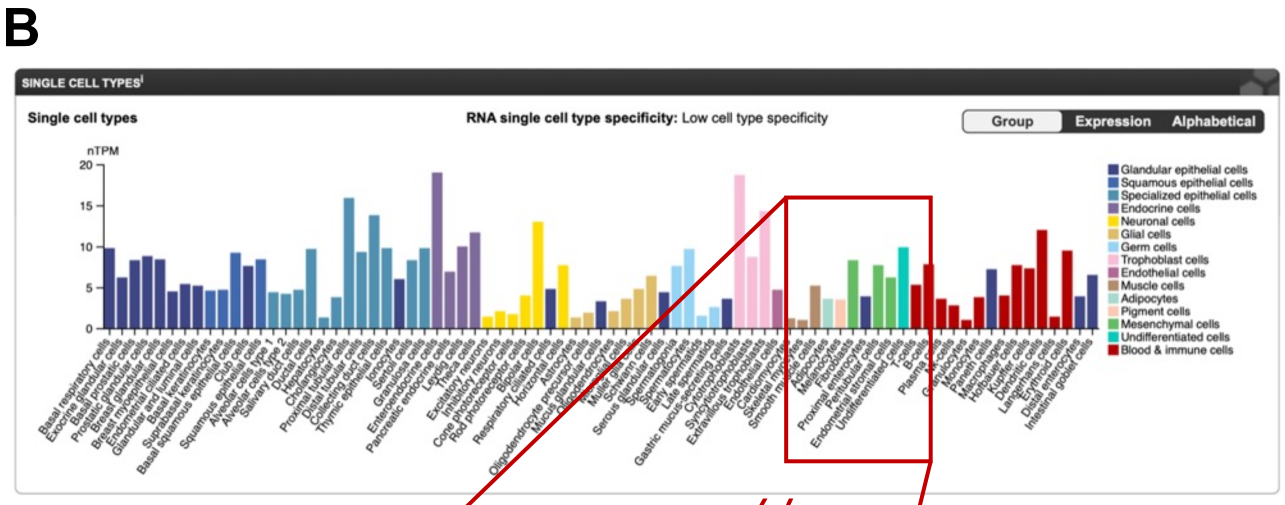

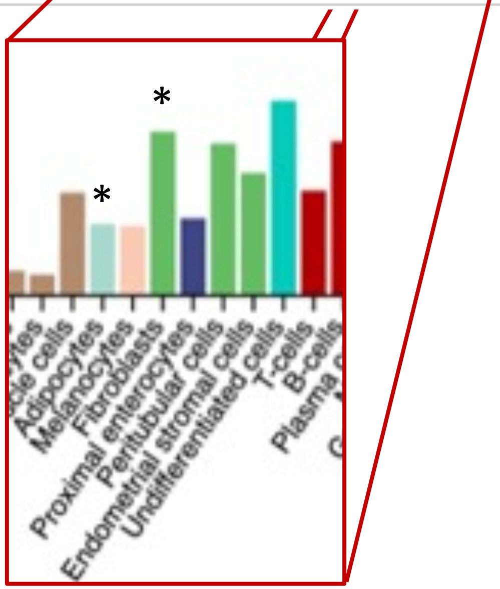

Supplementary Figure S4. PRMT6 expression in human.
Depicted are the results of the single cell analysis from the human genome atlas (date of generation: 16.05.2023). **(A)** Based on single cell sequencing, cell types are clustered according to their identity and expression levels of PRMT6. Blood & immune cells are shown in red, mesenchymal cells in green, smooth muscle cells in beige and adipocytes in mint green. The expression is given in the unit Transcripts per Million. Fibroblasts have a higher Prmt6 expression than adipocytes. Adipocytes are marked with red circle and arrow. **(B)** Prmt6 is not cell type specific as it occurs in a variety of single cell types. As can be seen in the enlarged section, human MSCs express more PRMT6 than adipocytes (marked with star).

Supplementary Figure 5
Related to Figure 1

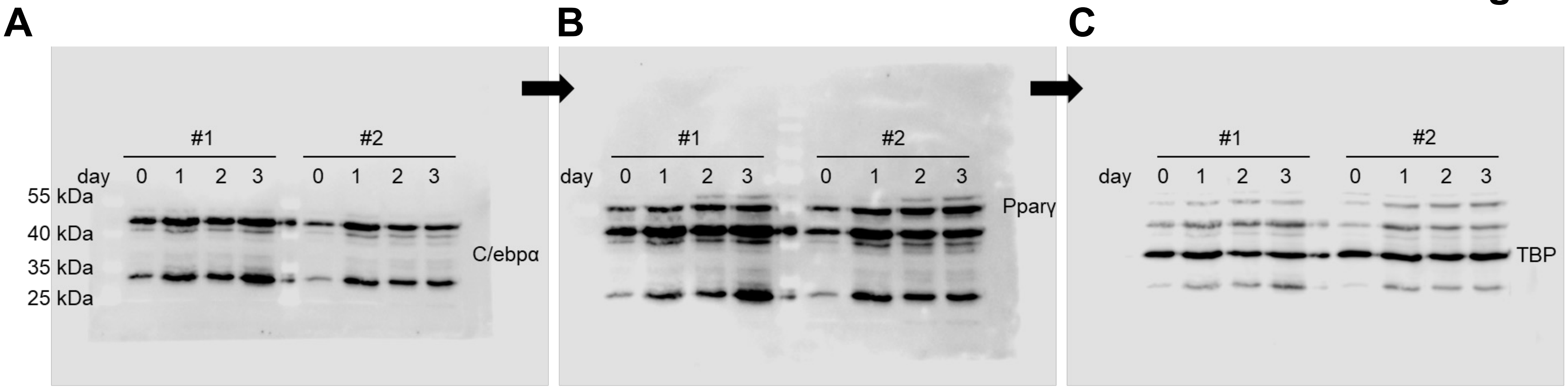

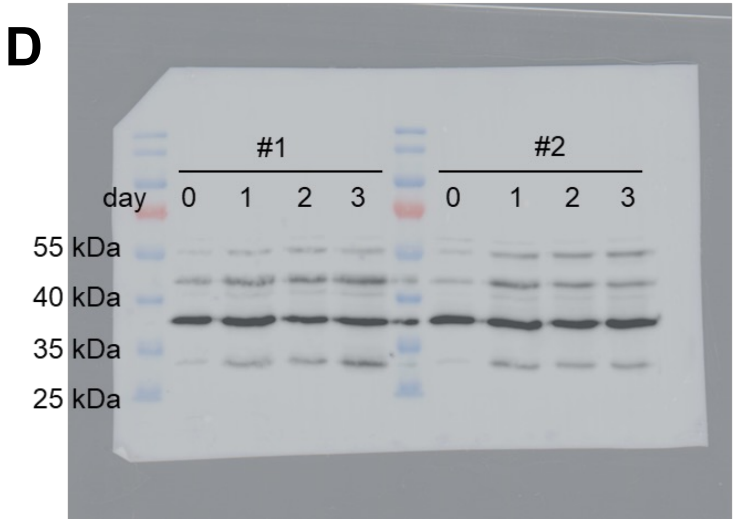

Supplementary Figure S5. Raw data of the Western Blot upon adipogenesis in ST2 cells.

(A-D) Raw data related to the Western Blot shown in **Figure 1D**. The time points are 0, 1, 2 and 3 days after adipocyte induction. **(A)** C/ebpα (Cell Signaling, #8178) and **(B)** Pparγ (Cell Signaling, #2443) were stained directly after each other. The membrane was not stripped in between different antibody stainings. **(C)** As a loading control TBP (Abcam, ab63766) was used. **(A-C)** All antibodies were used with a dilution of 1:1000. Secondary antibody anti-rabbit IgG H&L (HRP) (Abcam, ab97080) was used with a dilution of 1:10.000. All antibodies were diluted in 4% milk/TBS-T. For size estimation PageRuler™ (Invitrogen, 26617) was applied. **(D)** Overlay of the staining of TBP (C) with the colorimetric marker.

Supplementary Figure S6
Related to Figure 1

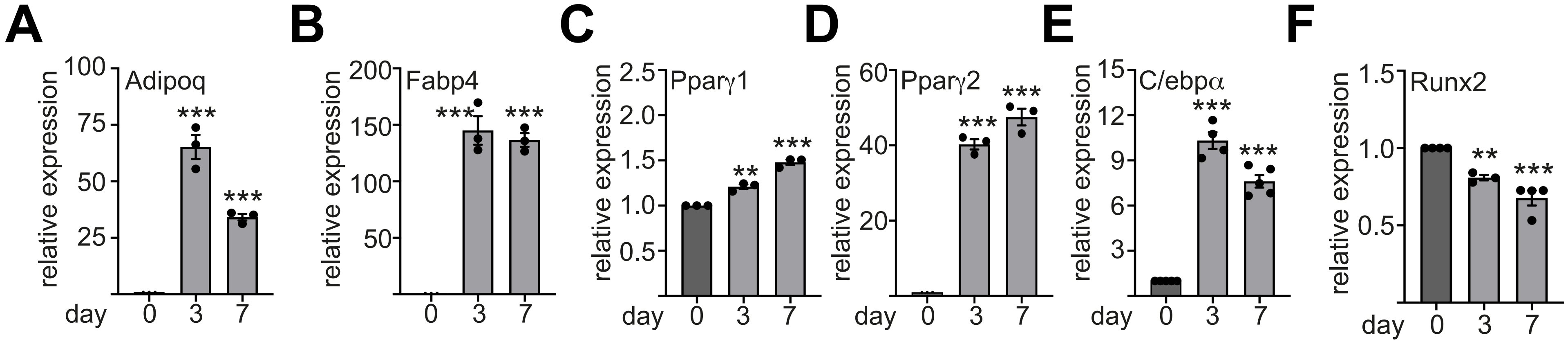

Supplementary Figure S6. Gene expression upon adipogenic differentiation in ST2 cells.
Gene expression was determined by qRT-PCR analysis of cells at the indicated time point upon differentiation. **(A-B)** Expression of the adipocyte specific genes **(A)** *Adipoq* and **(B)** *Fabp4* during differentiation at the mRNA level. Increased expression levels at day 3 and 7 upon differentiation induction indicate a successful adipocyte differentiation of the ST2 cells. **(C-E)** Expression of the early markers for adipogenesis *Pparγ1* **(C)**, *Pparγ2* **(D)** and *C/ebpα* **(E)** were determined. They show higher expression levels compared to undifferentiated cells at day zero. **(F)** The transcription factor Runx2 is relevant for osteogenesis. It is known to suppresses adipogenesis. *Runx2* shows lower mRNA expression levels upon adipogenic differentiation. **(A-F)** qRT-PCR was performed with gene specific primer pairs. Data are shown as relative expression normalized to the expression of the housekeeping gene *TBP*, values from day zero were set as one. The error bars display the standard error from the mean of three experiments. The *P*-values were calculated using ANOVA. $^{**}P < 0.002$, $^{***}P < 0.001$.

Supplementary Figure S7
Related to Figure 1

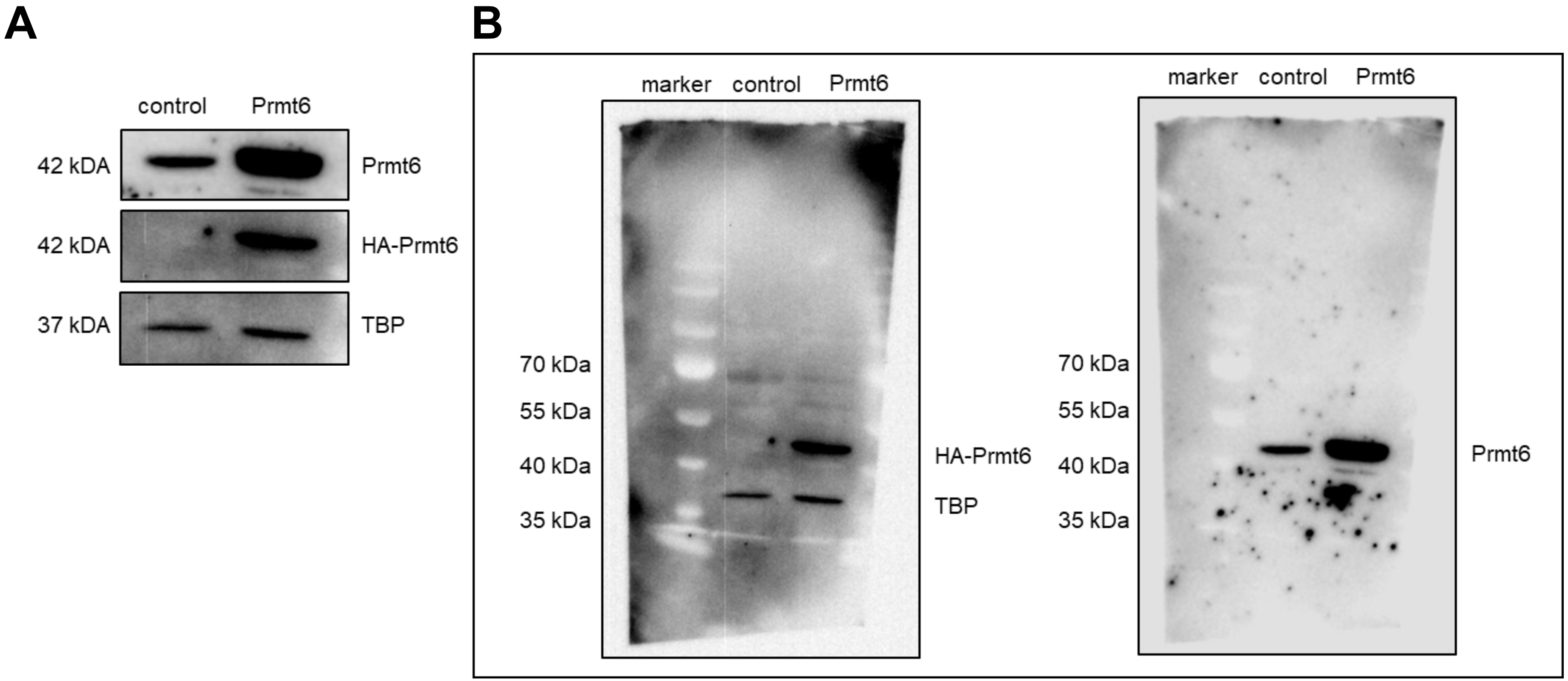

Supplementary Figure S7. Prmt6 overexpression.
(A) ST2 cells were lentiviraly transduced with either empty vector or HA-Prmt6-LeGOiG2 to overexpress Prmt6. Western Blot analysis indicates HA-Prmt6 expression. Prmt6 transduced cells show a strong staining for HA and an increased staining for Prmt6, indicating that the overexpression has worked. No HA-Prmt6 staining was detected in the control cells. Western Blot analysis with an TBP antibody served as loading control. **(B)** Raw data of the Prmt6 overexpression Western Blot. HA-Prmt6 was stained with HA.11 Clone 16B12 antibody (BioLegend, 901502) with a dilution of 1:1000. As a loading control TBP (Abcam, ab63766) was used with a dilution of 1:1000. After stripping, Prmt6 (Cell Signaling, #14641, 1:1000) was stained. Secondary antibodies anti-mouse IgG H&L (HRP) (Abcam, ab97040) and anti-rabbit IgG H&L (HRP) (Abcam, ab97080) were used with a dilution of 1:10.000. Antibodies were diluted in 4% milk/TBS-T. For size estimation PageRuler™ (Invitrogen, 26617) was applied.

Supplementary Figure S8

Related to Figure 1

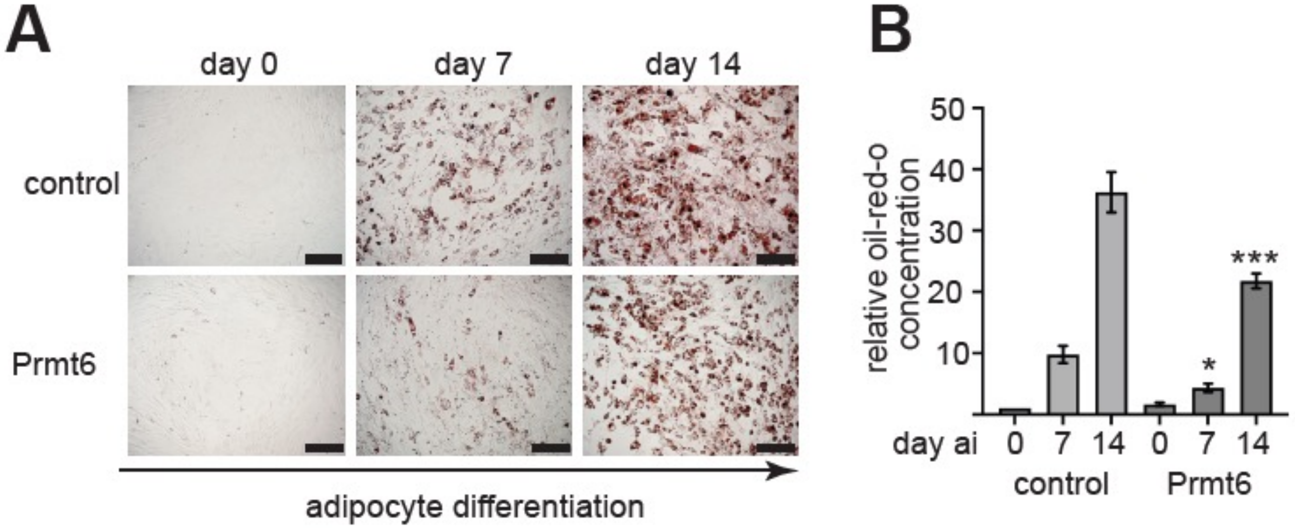

Supplementary Figure S8. Prmt6 overexpression represses adipocyte differentiation.

(A) Altered lipid droplet accumulation upon Prmt6 overexpression. Prmt6 was overexpressed in ST2 cells by a lentiviral expression system. Cells were subjected to adipocytic differentiation for the indicated time and stained for lipid formation by oil-red-o staining. The scale bar indicates 200 µm. **(B)** Relative oil-red-o concentration in the Prmt6 overexpressing cells compared to the control cells. The error bars display the standard error from the mean of three independent experiments. The P-values were calculated using ANOVA. $^{*}P < 0.033$, $^{***}P < 0.001$.

Supplementary Figure S9

Related to Figure 2

A

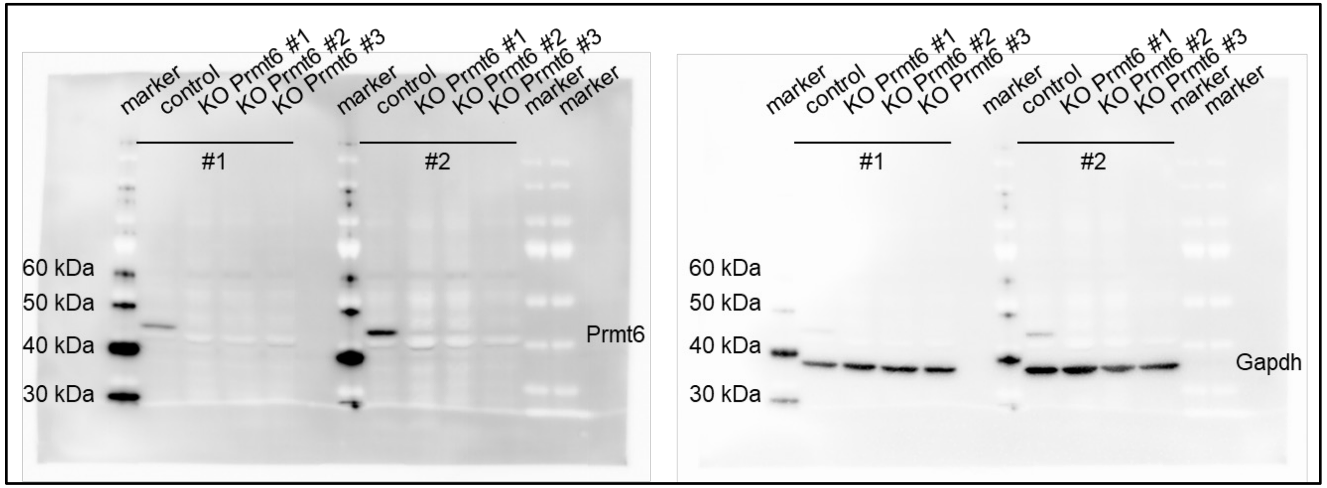

B

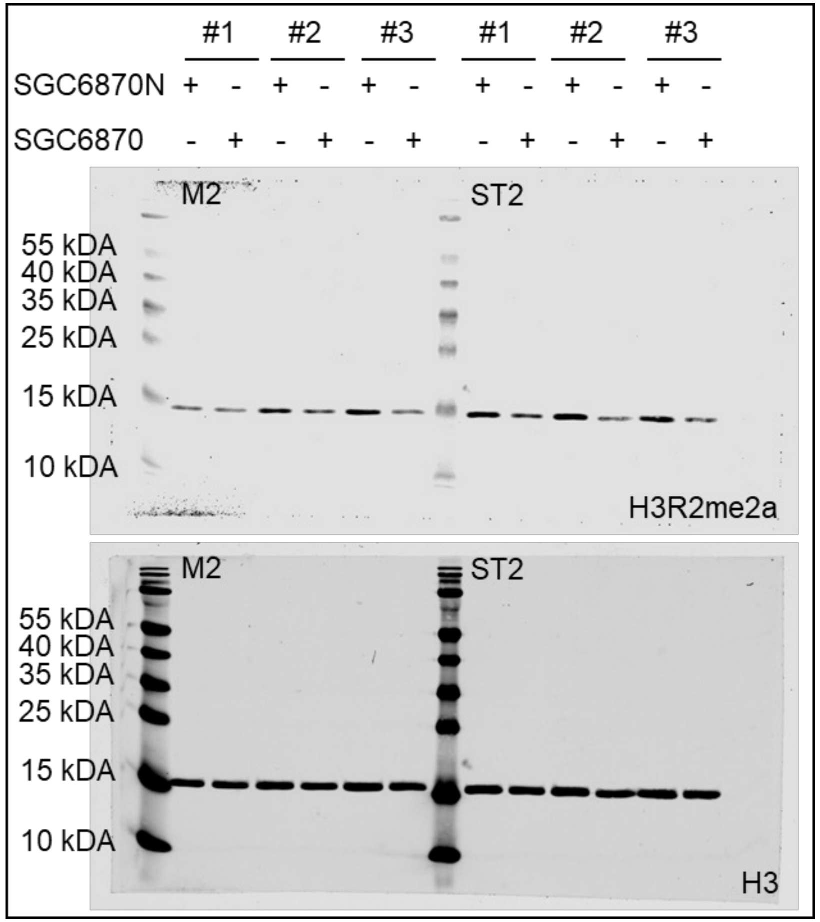

Supplementary Figure S9. Raw data of Prmt6 knockout.
(A) ST2 cells were lentiviraly transduced with gRNAs against Prmt6. CRISP/Cas9 mediated knockout was confirmed. Prmt6 was only detectable in the control cells. Western Blot was performed with 10 µg (#1) and 20 µg (#2) of whole cell lysates. As a marker MagicMark™ XP (Invitrogen, LC5602) was used. Prmt6 was stained with the antibody PRMT6 (D5A2) from Cell Signaling (#14641) with a dilution of 1:1000. As a loading control GAPDH (Invitrogen, MA5-15738) was used with a dilution of 1:1000. Secondary antibodies anti-mouse IgG H&L (HRP) (Abcam, ab97040) and anti-rabbit IgG H&L (HRP) (Abcam, ab97080) were used with a dilution of 1:10.000. All antibodies were diluted in 4% milk/TBS-T. **(B)** M2 and ST2 cells were treated with 5 µM of Prmt6 inhibitor SGC6870 or its inactive control SGC6870N. After 24h, adipogenesis was induced for 72h. Raw data of the Western Blot analysis upon Prmt6 inhibitor treatment or its inactive control compound during adipogenesis in M2 and ST2 cells. Histone extracts of three biological replicates were stained with H3R2me2a (Cell Signaling, #33725, 1:1000) and Histone H3 (Proteintech, 68345-1-lg,1:10.000) simultaneously. Secondary antibodies (Li-Cor, 926-68070 and 926-32211) were used at a dilution of 1:10.000. PageRuler™ (Invitrogen, 26617) was applied for size estimation. This blot was used to quantify the amount of H3R2me2a normalized to Histone H3.

Supplementary Figure S10
Related to Figure 2

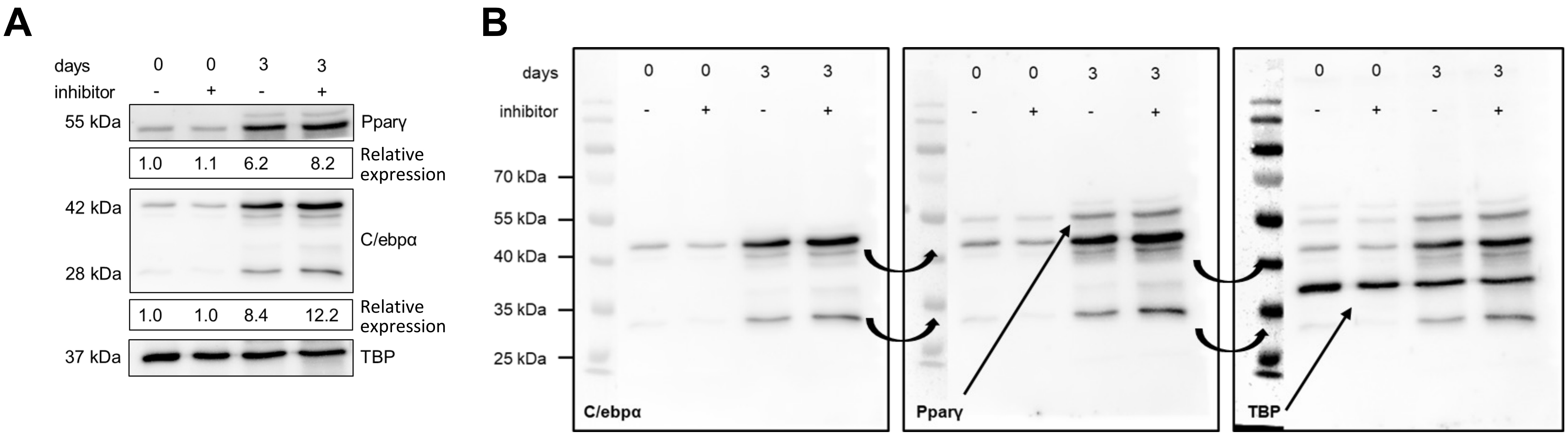

Supplementary Figure S10. Inhibition of Prmt6 promotes adipogenesis.
(A) ST2 cells were treated with 5 µM of Prmt6 inhibitor SGC6870 or its inactive control SGC6870N. After 24h, cells were induced with adipocyte differentiation media supplemented with inhibitor or control. Western Blot analysis indicates Pparγ and C/ebpα expression during adipocyte differentiation. Western Blot was performed with 20 µg lysates of differentiated ST2 cells at the indicated time points and antibodies against the shown proteins. Quantification values are listed underneath the corresponding Western Blot. The values indicate TBP normalised protein expression relative to day zero of control treated cells. No different protein levels of Pparγ were detected at day zero upon inhibition of Prmt6. Pparγ levels are increased in the Prmt6-inhibited cells compared to the control cells at day 3 after adipocyte induction. No different protein levels C/ebpα were detected at day zero upon inhibition of Prmt6. C/ebpα levels are increased in the Prmt6-inhibited cells compared to the control cells at day 3 after adipocyte induction. Western Blot with a TBP antibody served as a loading control. **(B)** Raw data for the previous Western Blot is shown. The Western Blot was stained with C/ebpα (Cell Signaling, #8178) and Pparγ (Cell Signaling, #2443) primary antibodies. As a loading control Tbp (Abcam, ab63766) was used. The membrane was not stripped in between different antibody stainings. All primary antibodies were used with a dilution of 1:1000. Secondary antibody anti-rabbit IgG H&L (HRP) (Abcam, ab97080) was used with a dilution of 1:10.000. All antibodies were diluted in 4% milk/TBS-T. For size estimation PageRuler™ (Invitrogen, 26617) was applied.

Supplementary Figure S11

Related to Figure 3

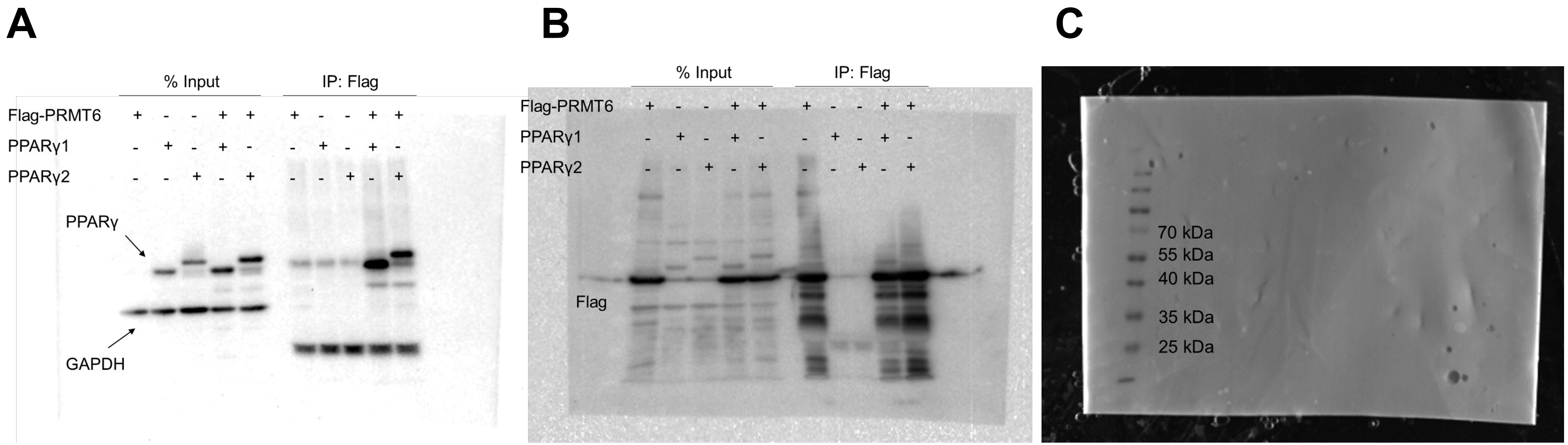

Supplementary Figure S11. Raw data of CoIP.
To determine the association of Prmt6 with Pparγ1 and Pparγ2, a coimmunoprecipitation (CoIP) was performed. Flag-tagged PRMT6 was expressed with isoforms of PPARγ. Anti-Flag beads (Thermo Scientific, A36797) were used to precipitate PRMT6. **(A)** Western Blot staining with PPARγ antibody showed that both isoforms coprecipitated with PRMT6. PPARγ was stained with the antibody PPARγ (81B8) from Cell Signaling (#2443) with a dilution of 1:1000. **(B)** PRMT6 was stained with the Flag antibody (Sigma-Aldrich, F7425). Image shows adjusted background. As a loading control for the input GAPDH (Invitrogen, MA5-15738) was used. All antibodys were used with a dilution of 1:1000. Secondary antibodies anti-rabbit IgG H&L (HRP) (Abcam, ab97080) and anti-mouse IgG H&L (HRP) (Abcam, ab97040) were used with a dilution of 1:10.000. Antibodies were diluted in 4% milk/TBS-T. **(C)** As a marker PageRuler™ (Invitrogen, 26617) was used.

Supplementary Figure S12

Related to Figure 3

A

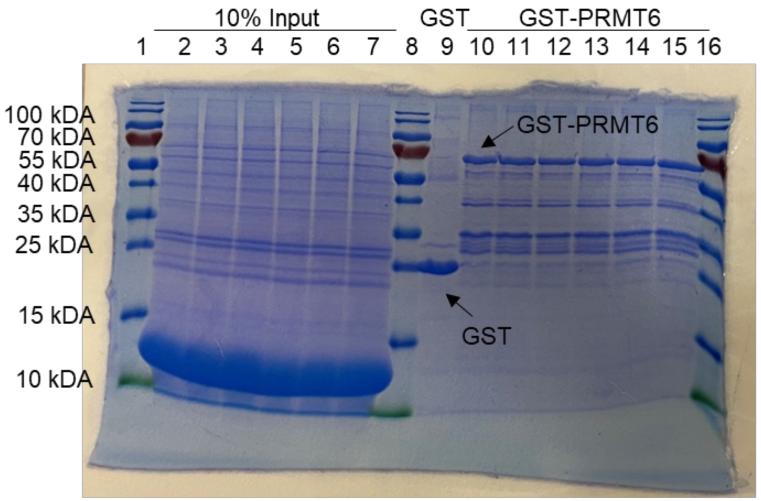

1) PAGE Ruler Prestainded
2) hPPARG 1-505aa (full length)
3) hPPARG 1-140aa
4) hPPARG 136-265aa
5) hPPARG 259-376aa
6) hPPARG 370-505aa
7) hPPARG 30-140aa
8) PAGE Ruler Prestainded
9) hPPARG 1-505aa (full length) GST
10) hPPARG 1-505aa (full length) GST-PRMT6
11) hPPARG 1-140aa GST-PRMT6
12) hPPARG 136-265aa GST-PRMT6
13) hPPARG 259-376aa GST-PRMT6
14) hPPARG 370-505aa GST-PRMT6
15) hPPARG 30-140aa GST-PRMT6
16) PAGE Ruler Prestainded

B

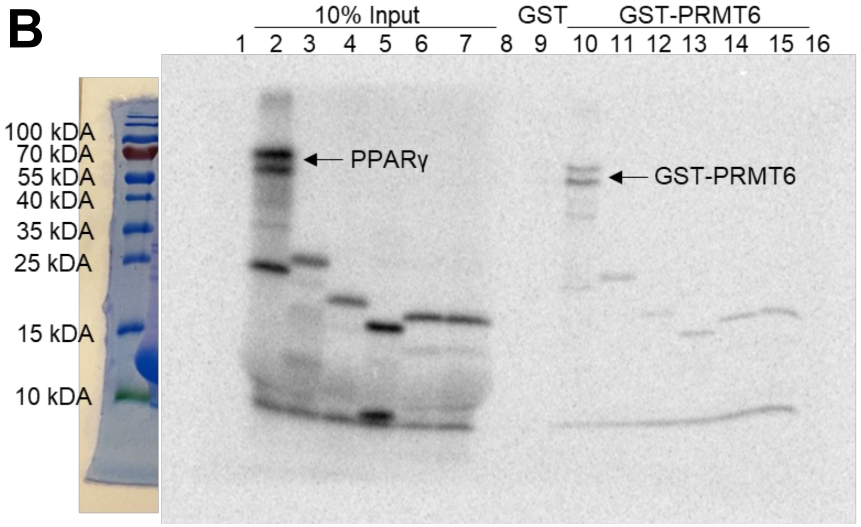

Supplementary Figure S12. Raw data of GST pulldown.
To further confirm the interaction of Prmt6 and Pparγ, a GST pulldown with PPARγ deletion constructs was done. Herefore, GST-PRMT6 and in vitro translated S^{35} labeled PPARγ was used. **(A)** Coomassie staining visualized the proteins on the gel. As a marker PageRuler™ (Invitrogen, 26617) was applied. **(B)** Radioactive detection showed PPARγ in the input line as well as in the GST pulldown.

Supplementary Figure S13

Related to Figure 5

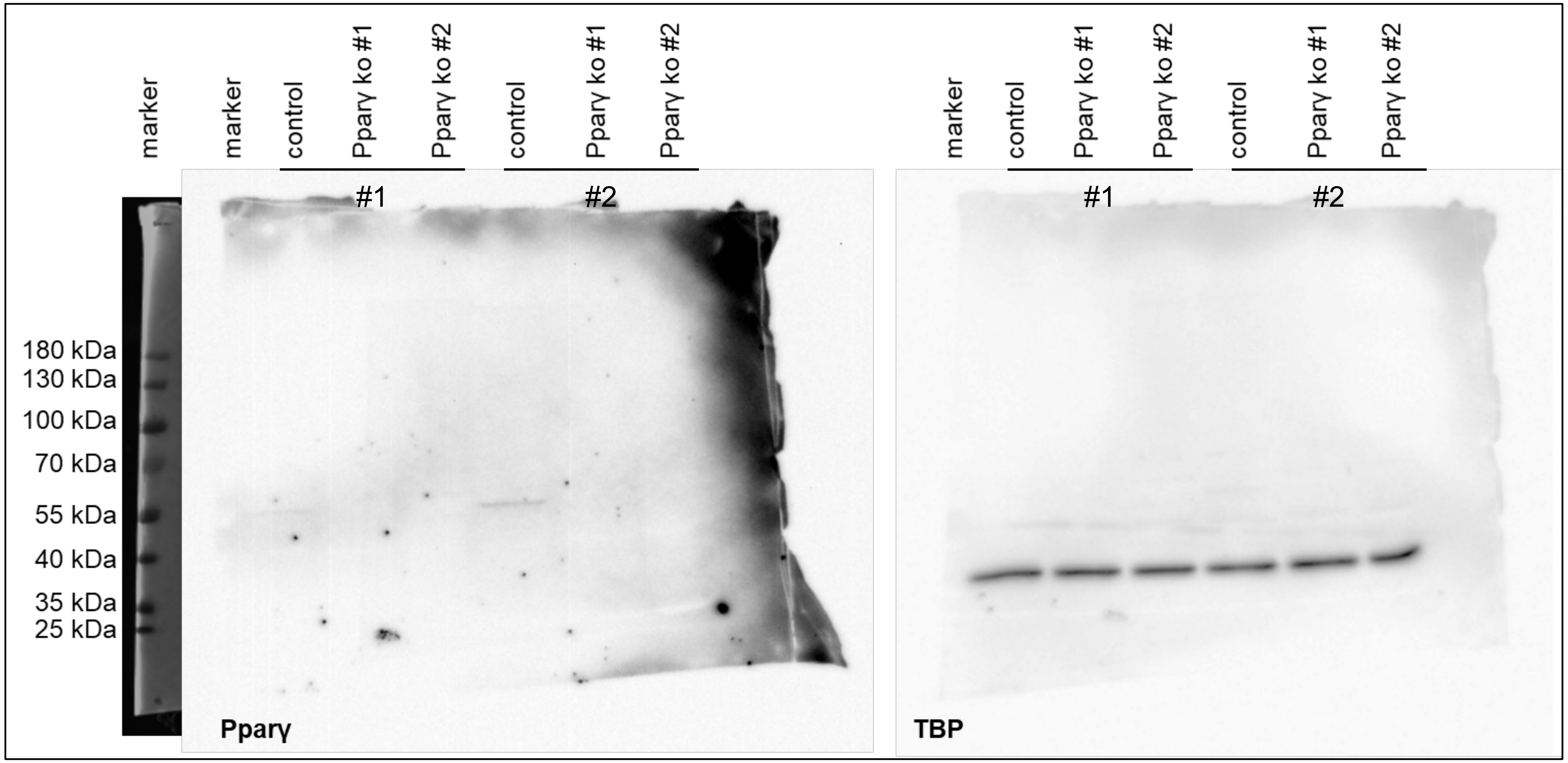

Supplementary Figure S13. Raw data of Pparγ knockout.
ST2 cells were lentiviraly transduced with two gRNAs against Pparγ and a non-targeting gRNA as a control. CRISPR/Cas9 mediated knockout was confirmed in a Western Blot. Pparγ was detected in the control cells. No Pparγ was found in the knockout cell lines. Western Blot was performed with 30 µg of nuclear extracts. As a marker PageRuler™ (Invitrogen, 26617) was used. Pparγ was stained with the antibody PPARγ (81B8) from Cell Signaling (#2443) with a dilution of 1:1000. As a loading control TBP (Abcam, ab63766) was used with a dilution of 1:1000. Secondary antibody anti-rabbit IgG H&L (HRP) (Abcam, ab97080) was used with a dilution of 1:10.000. Antibodies were diluted in 4% milk/TBS-T. For the figure, the contrast and the sharpness of the TBP-stained blot were adjusted.
