# Supplementary material for: Prmt6 represses the pro-adipogenic Ppar-gamma–C/ebp-alpha transcription factor loop
Source: Sci Rep. 2024 Mar 20;14:6656. doi: 10.1038/s41598-024-57310-9 (PMC10954715; doi:10.1038/s41598-024-57310-9)
Supplement: Supplementary file 2 — Supplementary Tables. [file 41598_2024_57310_MOESM2_ESM.docx]

Supplementary Material

Gerstner et al.

Table 1: Cloning primers

| Name | Forward sequence 5' →3' | Reverse sequence 5' →3' | Purpos |
| --- | --- | --- | --- |
| mPRMT6_EcoRI_HA-Tag | AAAGAATTCATGTACCCATACGATGTTCC  AGATTACGCTGCATCGCTGAGCAAGAAAAGA | AAAGCGGCCGCTCAGTCCTCCATGGCAAAGTC | mPRMT6 with HA-Tag in LeGOiG2 |
| gRNA_non_targeting_mouse | CACCGAGATTCGATACTGCGGTCA | AAACTGACCGCAGTATCGAATCTC | guide RNA cloning into lentiCRISPRV2 |
| gRNA_mPrmt6_1 | CACCGTTTCAAGTGACCTTTCCCGG | AAACCCGGGAAAGGTCACTTGAAAC | guide RNA cloning into lentiCRISPRV2 |
| gRNA_mPrmt6_2 | CACCGCACCGGCTCGTTCAAGTAG | AAACCTACTTGAACGAGCCGGTGC | guide RNA cloning into lentiCRISPRV2 |
| gRNA_mPparg_1 | CACCGAGTTAGAAGGTTCTTCATG | AAACCATGAAGAACCTTCTAACTC | guide RNA cloning into lentiCRISPRV2 |
| gRNA_mPparg_2 | CACCGCCATTGAGTGCCGAGTCTG | AAACCAGACTCGGCACTCAATGGC | guide RNA cloning into lentiCRISPRV2 |
| hPPARG 1-505aa | GCAGCGGCCGCAGGTGAAACTCTGGGAGAT | TGCTCTAGACTAGTACAAGTCCTTGTAGATC | cloning hPPARG into pcDNA3 (2HA) |

Table 2: Real-time PCR primers

| Name | Forward sequence 5' →3' | Reverse sequence 5' →3' |
| --- | --- | --- |
| Tbp | AGCTCTGGAATTGTACCGCA | TGACTGCAGCAAATCGCTTG |
| Prmt6 | AAACCTCTGGTGCTGTCCAC | CCGGCTCGTTCAAGTAGAGG |
| Pparγ2 | TTCGCTGATGCACTGCCTAT | GGAATGCGAGTGGTCTTCCA |
| Pparγ1 | CGAGTGTGACGACAAGGTGA | ACCGCTTCTTTCAAATCTTGTCTG |
| Pparγ total | TGTGAGACCAACAGCCTGAC | TCAGTGGTTCACCGCTTCTT |
| C/ebpα | CCGTGGTGGTTTCTCCTTGA | TTTTTGCTCCCCCTACTCGG |
| Adiponectin | TGACGACACCAAAAGGGCTC | CACAAGTTCCCTTGGGTGGA |
| Fabp4 | TCATAACCCTAGATGGCGGG | GCCTTTCATAACACATTCCACC |
| Adipsin | CCTACATGGCTTCCGTGCAA | CACCTGCACAGAGTCGTCAT |
| Runx2 | GCTTCATTCGCCTCACAAAC | TTAAACGCCAGAGCCTTCTTG |

Table 3: real-time PCR TaqMan probes

| Name | Assay ID | Supplier |
| --- | --- | --- |
| Prmt6 | Mm00619134_m1 | Invitrogen |
| Tbp | Mm00446973_m1 | Invitrogen |
| Pparγ | Mm01184322_m1 | Invitrogen |
| C/ebpα | Mm00514283_s1 | Invitrogen |
| Fabp4 | Mm00445878_m1 | Invitrogen |
| Aipoq | Mm00456425_m1 | Invitrogen |

Table 4: ChIP primers

| Name | Forward sequence 5' →3' | Reverse sequence 5' →3' |
| --- | --- | --- |
| *Pparγ* prom | CATGGACATCGGTCTGAGGG | CCGCCTTGCTCCTCACAG |
| Pparγ control region | TACAGAGAACACAGTAGTGAGGTA | GCACTCTACTGACTGACTTACCC |
| *C/ebpα* prom | CTCACCGCCTTGGAAAGTCA | TGTCCAAACGGGTCTCGGAT |
| C/ebpα control region | CGAAGTGGTGTTGAACCCGT | AGAGTTCTTGCCTGACGACTT |

Table 5: Primary antibodies

| Target | Cat.No. | Supplier | Dilution |
| --- | --- | --- | --- |
| β-Actin (8H10D10) | 3700 | Cell Signaling | 1:1500 |
| β2-microglobulin | 59035 | Cell Signaling | 1:1000 |
| C/EBPα (D56F10) | 8178 | Cell Signaling | 1:1000 |
| FABP4 | 2120 | Cell Signaling | 1:1000 |
| Flag | F7425 | Sigma-Aldrich | 1:1000 |
| GAPDH loading Control | MA5-15738 | Invitrogen | 1:1000 |
| HA.11 Clone 16B12 | 901502 | BioLegend | 1:1000 |
| PPARγ (81B8) | 2443 | Cell Signaling | 1:1000 |
| PRMT6 (D5A2N) | 14641 | Cell Signaling | 1:1000 |
| TATA binding Protein | ab63766 | Abcam | 1:1000 |
| Histone H3 | 68345-1-lg | Proteintech | 1:10000 |
| H3R2me2a | 33725 | Cell Signaling | 1:1000 |

Table 6: Secondary antibodies

| Target | Cat.No. | Supplier | Dilution |
| --- | --- | --- | --- |
| anti-mouse IgG H&L (HRP) | ab97040 | Abcam | 1:10000 |
| anti-rabbit IgG H&L (HRP) | ab97080 | Abcam | 1:10000 |
| anti-mouse IgG IRDye® 680RD | 926-68070 | Li-Cor | 1:10000 |
| anti-rabbit IgG (H& L) IRDye 800CW | 926-32211 | Li-Cor | 1:10000 |

Table 7: ChIP antibodies

| Target | Cat.No. | Supplier | ChIP |
| --- | --- | --- | --- |
| PRMT6 | 720142 | Invitrogen | Mix  2µg + 2µg |
| PRMT6 (Middle Region) | ABIN2778658 | antikörper-online.de |  |
| Rabbit IgG Isotype Control | 02-6102 | Invitrogen | 2µg/4µg |
| PPARγ (81B8) | 2443 | Cell Signaling | 1:100 |
| CBP | PA5-27369 | Invitrogen | 2.5 µg |
| HDAC1 | PA1-860 | Invitrogen | 2.5 µg |
| Histone H3 | PA5-49579 | Invitrogen | 2 µg |
| H3R2me2a | ab175007 | Abcam | 2 µg |
| H3K4me3 | ab8580 | Abcam | 2 µg |
| RNA polymerase II CTD repeat YSPTSPS (phospho S2) | ab193468 | Abcam | 4 µg |
| Rpb1 CTD (4H8) | 2629 | Cell Signaling | 10 µl |

Table 8: Antibodies for immunofluorescence

| Target | Cat.No. | Supplier | Staining |
| --- | --- | --- | --- |
| Phalloidin-iFluor 555 | ab176756 | Abcam | 1:2000 |
| DAPI (4',6-Diamidino-2-phenylindole Dihydrochlorid) | D8417 | Sigma-Aldrich | 1:5000 |
| PRMT6 | 720142 | Invitrogen | 1:250 |
| Goat anti-Rabbit IgG (H+L) Secondary Antibody, Alexa Fluor™ Plus 488 | A32731 | Invitrogen | 1:1000 |

Table 9: Additives for adipocyte induction media

| Substance | Cat.No. | Supplier |
| --- | --- | --- |
| Dexamethasone | D4902 | Sigma-Aldrich |
| IBMX | sc-201188 | Santa Cruz Biotechnology |
| Rosiglitazone | R2408 | Sigma-Aldrich |
| Insulin, human recombinant, zinc solution | 12585014 | Gibco |

Table 10: Inhibitor compounds

| Substance | | Cat.No. | Supplier |
| --- | --- | --- | --- |
| SGC6870 | PRMT6 inhibitor | SML2760 | Sigma-Aldrich |
| SGC6870N | control compound | SML2761 | Sigma-Aldrich |
